# Investigating the Role of Diet-Manipulated Gut Bacteria in Pathogenesis of Type 2 Diabetes Mellitus—An *In Vitro* Approach

**DOI:** 10.3390/nu18020279

**Published:** 2026-01-15

**Authors:** Asha Guraka, Marie Lush, Georgios Zouganelis, Joe Waldron, Subbareddy Mekapothula, Jinit Masania, Gareth Wynn Vaughan Cave, Myra Elizabeth Conway, Gyanendra Tripathi, Ali Kermanizadeh

**Affiliations:** 1 College of Science and Engineering, University of Derby, Derby DE22 1GB, UK; 2 School of Science and Technology, Nottingham Trent University, Nottingham NG11 8NS, UK

**Keywords:** gut microbiome, type 2 diabetes mellitus, *in vitro* approach, *Bacteriodes thetaiotaomicron*, Lactobacillus fermentum, diet, short-chain fatty acids

## Abstract

**Background:** The human gut microbiome is highly complex, and its composition is strongly influenced by dietary patterns. Alterations in microbiome structure have been associated with a range of diseases, including type 2 diabetes mellitus. However, the underlying mechanisms for this remain poorly understood. In this study, a novel *in vitro* approach was utilized to investigate the interplay between gut bacteria, dietary metabolites, and metabolic dysfunction. **Methods:** Two representative gut bacterial species—*Bacteroides thetaiotaomicron* and *Lactobacillus fermentum*—were isolated from human faecal samples and subjected to controlled dietary manipulation to mimic eubiotic and dysbiotic conditions. Metabolites produced under these conditions were extracted, characterized, and quantified. To assess the functional impact of these metabolites, we utilized the INS-1 832/3 insulinoma cell line, evaluating insulin sensitivity through glucose-stimulated insulin secretion and ERK1/2 activation. **Results:** Our findings demonstrate that metabolites derived from high-carbohydrate/high-fat diets exacerbate metabolic dysfunction, whereas those generated under high-fibre conditions significantly enhance insulin secretion and glucose-dependent ERK1/2 activation in co-culture compared to monocultures. **Conclusions:** This work systematically disentangles the complex interactions between gut microbiota, diet, and disease, providing mechanistic insights into how microbial metabolites contribute to the onset of metabolic disorders.

## 1. Introduction

The human gut harbours over 100 trillion microbial cells that maintain a symbiotic relationship with the host, playing essential roles in physiology, metabolism, nutrition, and immune regulation [1]. Consequently, the gut microbiome is often regarded as a microbial “endocrine organ” The dominant bacterial phyla include *Bacillota* (*Firmicutes*; 60–80%) and *Bacteroidota* (*Bacteroidetes*; 20–30%) [1,2,3,4,5].

Members of these phyla ferment indigestible dietary fibres, complex carbohydrates, proteins, and other macronutrients, producing short-chain fatty acids (SCFAs)—small metabolites (~1500 Da) such as acetate, butyrate, and propionate [6,7]. SCFAs are critical for gut homeostasis and activate signalling pathways including cAMP/PKA and MAPK/ERK, while increasing levels of glucagon-like peptide-1 (GLP-1) and peptide YY (PYY). These actions enhance glucose metabolism, adipogenesis, and insulin sensitivity [8,9].

SCFAs also maintain gut barrier integrity by stimulating AMPK-mediated mucin and antimicrobial peptide production, strengthening epithelial tight junctions and preventing translocation of bacterial toxins. Furthermore, SCFAs exhibit anti-inflammatory properties by inhibiting histone deacetylase activity [10,11,12,13]. Dysbiosis triggered by dietary changes, antibiotics, environmental stress, or sedentary lifestyle disrupts SCFA balance and contributes to metabolic disorders such as type 2 diabetes mellitus (T2DM) and progressive liver disease [1,7]. Notably, high-fat, low-fibre diets exacerbate dysbiosis by altering *Bacillota* and *Bacteroidota* composition [14,15,16,17,18].

T2DM is a multifactorial, polygenic disease characterized by impaired insulin secretion and insulin resistance, accounting for ~90% of global diabetes cases among adults aged 20–79 [19,20,21,22]. Current methodologies for studying microbiome–disease interactions, include rodent models and epidemiological studies which are extremely useful informative but are limited by interspecies differences and confounding factors such as sex, lifestyle, and diet [23,24,25].

To address these limitations, this study introduces a novel *in vitro* approach to examine the impact of dietary variation on gut bacterial composition and its downstream effects on T2DM onset. The workflow involved the isolation of key gut bacterial species from human faecal samples, bacterial manipulation under controlled dietary conditions, and characterization of the bacterial produced metabolites. Finally, the influence of these diet-modulated metabolites on glucose metabolism and insulin sensitivity was assessed using a T2DM-relevant insulinoma cell line.

## 2. Methods

### 2.1. Human Faecal Sample Collection

Fresh human faecal samples were obtained from healthy individuals aged 18–60 with no history of antibiotic use in the last three months (Arden Tissue Bank, University Hospitals Coventry and Warwickshire NHS Trust) under NHS ethical approval. The research was conducted with approval from the Health and Safety Executive (HSE) Biosafety and Microbiology Containment, permitting work with Class 2 bacteria, as outlined on the HSE biosafety website (https://www.hse.gov.uk/biosafety/ (accessed on 3 December 2025)).

### 2.2. Isolation and Identification of Gut Bacteria

The faecal samples (1 g) were homogenized in pre-reduced phosphate-buffered saline (PBS) in an anaerobic chamber (Don Whitley Scientific, Bingley, UK) to maintain anoxic conditions for gut bacteria viability. Six-fold serial dilutions were prepared, and 100 µL aliquots from each dilution were spread onto pre-reduced agar plates, including fastidious anaerobe agar (FAA) with Neomycin and Aztreonam, and 7% horse blood (HB) (E&O Labs, Bonnybridge, UK), along with selective media including De Man, Rogosa, and Sharpe (MRS) and brain heart infusion (BHI) agar (OXOID, Basingstoke, UK). The plates were incubated at 37 °C for 48 h in an anaerobic chamber to promote specific gut bacterial colonies [26]. For identification, PCR targeting the V3–V4 hypervariable regions of 16S rRNA genes was performed [27,28,29], followed by Sanger sequencing to identify species, resulting in *Bacteriodes thetaiotaomicron BFG109* (*B. thetaiotaomicron*) and *Lactobacillus fermentum L4* (*L. fermentum*) [26] (Supplementary Figure S1).

### 2.3. Bacterial Culture

The identified bacterial strains, *B. thetaiotaomicron* and *L. fermentum*, were isolated via pure inoculation under anaerobic conditions at 37 °C on FAA agar supplemented with 7% HB (E&O Labs). The monocultures were cultured using 200 mL fastidious anaerobe (FAA) broth at 37 °C, 120 RPM (E&O Labs), a medium optimised for the growth of anaerobic organisms, which contained essential growth factors such as vitamin K (0.0005 g/L) and haemin (0.005 g/L), essential to support the proliferation of specific anaerobes. Additionally, sodium thioglycolate (0.5 g/L) and L-cysteine HCl (0.5 g/L) were added to lower the pH and maintain a reducing environment. Moreover, resazurin (0.001 g/L) was added to act as a redox indicator, while sodium chloride (2.5 g/L) helped in the maintenance of osmotic balance.

The co-culture of the isolated bacteria was cultured following an optimised protocol [26] under controlled environmental conditions using a direct mixing method in FAA broth and in anaerobic conditions at 37 °C. The bacterial concentrations of monocultures were normalised with the inoculation ratio of 1:2 of *B. thetaiotaomicron* and *L. fermentum*. To facilitate the retrieval of a representative metabolome and secretome, growth curves for each bacterium were monitored at multiple time points by measuring optical density at 600 nm (OD600) and colony-forming units per millilitre (CFU/mL) to ensure an accurate assessment of growth, allowing the cultures to reach optimal-log phase, which is critical for maximising the extraction of metabolites, proteins and enzymes for downstream analysis.

### 2.4. Manipulation of Gut Bacteria Under Different Dietary Conditions In Vitro

After the optimization of the growth conditions and concentration range of the bacterium, the individual strains of *B. thetaiotaomicron* and *L. fermentum* were inoculated in 100 mL of FAA broth before manipulation using varying dietary conditions. The supplements involved preparing a stock solution of 1 M low-viscosity sodium carboxymethyl cellulose (CMC) in 2 mL of autoclaved warm distilled water to dissolve, which was then added to 100 mL of FAA broth medium to achieve a final concentration of 20 mM, referred to as high-fibre. Alternatively, a combination of 1 mL stock of 1 M D ± glucose (Sigma-Aldrich, Gillingham, UK) and approximately100 mM low-density lipoprotein (LDL) in 1.5 mL stock (Lee Biosolutions, Maryland Heights, MO, USA) was used, resulting in final concentrations of 10 mM D ± glucose and 1.5 mM LDL in 100 mL of FAA broth medium, referred to as high-carbohydrate/fat. Moreover, the FAA broth without dietary supplementation served as the control.

The monocultures of *B. thetaiotaomicron* and *L. fermentum* were incubated anaerobically at 37 °C for 24 h using these diets. Additionally, the co-culture of bacteria was cultured under the same conditions. The influence of diet on growth and understanding the metabolic inputs based on the optimal-log phase bacterial growth was assessed after five-fold serial dilutions and plating on FAA agar supplemented with 7% horse blood (HB), followed by quantification of colony-forming units per millilitre (CFU/mL).

### 2.5. Metabolite Extraction and Untargeted Characterization

The metabolite extraction protocol was optimized to allow the identification and quantification of key biochemical compounds, including SCFAs, amino acids, lipids, and enzyme cofactors produced by bacteria during catabolic and anabolic pathways [26]. Firstly, untargeted metabolomics was performed using *m*/*z* data acquisition for metabolites from the bacteria. The characterization was conducted using an Agilent 8860 gas chromatography (5977B) mass spectrometry (GC-MS) system (Agilent Technologies Inc., Santa Clara, CA, USA), equipped with an inbuilt National Institute of Standards and Technology (NIST) database and single quadrupole for metabolite analysis.

### 2.6. Quantitative GC-MS Analysis

The sample analysis was performed using Agilent 8860 gas chromatography system coupled with a single quadrupole Agilent 5977B mass spectrometry (GC-MS) with an inbuilt NIST database (Agilent Technologies Inc.) for direct analysis of metabolites of monocultures and their co-culture extracted after dietary manipulation.

The quantification was carried out using Selected Ion Monitoring (SIM) mode with a dwell time of 50 ms by utilizing the Agilent J&W DB-FFAP (30 m × 0.25 mm, 0.25 µm, 7-inch cage), which is a nitro terephthalic acid-modified polyethylene glycol (PEG) column (Agilent Technologies Inc.). The DB-FFAP column effectively quantifies SCFAs due to its high polarity and affinity for polar compounds such as fatty acids. The modified polyethene glycol stationary phase, with nitro terephthalic acid, boosts interactions with SCFAS free acidic groups through hydrogen bonding and acid-base interactions, leading to significant SCFA retention and ensuring efficient separation and quantification of these volatile fatty acids without derivatization [30,31]. Here, a total of 1 µL of sample was injected in splitless mode, which was further separated through the DB-FFAP column in which the initial GC oven temperature was increased from 50 °C to 250 °C at the rate of 4 min, followed by 25 °C/min to 200 °C, 15 °C/min to 250 °C and then held for 5 min. Helium gas (purity > 99%) was used as a carrier gas at a 1.2 mL/min flow rate with a total run time of 18.33 min. Overall, the energy of electron ionization was set to 70 eV.

Before quantification, MS data for standard analytes (acetic, propionic, butyric acids) and internal standard 2-ethyl butyric acid, in 80% methanol, were detected in the PEG column from *m*/*z* 20 to 500 Amu. The compounds were identified by comparing MS spectra with *m*/*z* values and retention time (selected ion monitoring (SIM) targeted ions of interest for quantification) [30]. The analytical standards included acetic acid (Cat no. 695092, Sigma-Aldrich), propionic acid (Cat no. 94425, Sigma-Aldrich), butyric acid (Cat no. 19215, Sigma-Aldrich), and internal standard, 2-ethyl butyric acid (Cat no. 109959, Sigma-Aldrich). The SCFA standards were dissolved in 80% methanol before being concentrated in nitrogen (N_2_) gas to eliminate the solvent and prepared for reconstitution. The concentrated residues were reconstituted in hexane for gas chromatography (GC) injection. The standards were analyzed in triplicate to plot calibration curves, using the response ratio by dividing the SCFA peak area by the internal standard peak area. The SCFA concentrations were quantified using SIM mode and the peak response ratio and linearity formula applied to the standards.

### 2.7. Endotoxin Quantification

A subset of the extracted metabolites was subjected to endotoxin removal to ensure the samples were LPS free. The endotoxins were removed using a high-efficiency endotoxin removal kit, Pierce™ High-Capacity Endotoxin Removal Spin 0.25 mL Columns (Thermo Fisher Scientific, Loughborough, UK), following the manufacturer’s protocol. Briefly, the samples were incubated with the endotoxin removal resin at a ratio of 0.25 mL/1 mL sample and gently mixed for 2 h at 4 °C. After the procedure, endotoxin levels were quantified using Pierce™ Chromogenic Endotoxin Quant Kit (Thermo Fisher Scientific). This highly sensitive test contains Limulus amebocyte lysate to detect endotoxins based on a colorimetric reaction. The samples were compared against a standard endotoxin curve, and absorbance was measured at 405 nm to quantify the endotoxin concentration in endotoxin units (EU/mL).

### 2.8. Mammalian Cell Culture

The humanised insulinoma cell line (INS-1 832/3) were a kind gift from Nottingham Trent University. The cells were cultured in RPMI 1640 medium (Sigma-Aldrich) supplemented with 10% foetal bovine serum (FBS), 1 mM sodium pyruvate, 10 mM HEPES, 50 µM β-mercaptoethanol, 2 mM L-glutamine, and 1% penicillin-streptomycin. The cells were incubated at 37 °C in a humidified atmosphere containing 5% CO_2_.

### 2.9. Diet-Manipulated Metabolite Exposure

The impact of different metabolites on β-cell viability and insulin production was undertaken. The INS-1 832/3 cells were seeded at 20,000 cells/well in 96-well plates for 24 h. As described above, the extracted metabolites from the mono- or the co-cultures following dietary manipulation were prepared (including samples with endotoxin (ET+) and following endotoxin removal (ET−). The concentrations of these metabolites were quantified using the Bicinchoninic Acid (BCA) assay (Thermo Fisher Scientific) before exposure to INS-1 832/3 cells at a concentration range of 125–1000 µg/mL.

### 2.10. Cytotoxicity Assessment

The Alamar Blue assay was used to evaluate the cytotoxicity of metabolites at 24 and 48 h. The cytotoxicity experiments included negative (complete cell culture medium) and positive controls (RIPA lysis buffer 1% *v*/*v*). Following the metabolite exposure, a stock concentration of 1 mg/mL resazurin sodium salt (Sigma-Aldrich) was diluted 1:10 in complete medium and added to cells for 2 h at 37 °C. The fluorescence was measured at excitation at 560 nm and emission at 590 nm using a Fluostar Omega plate reader (BMG Labtech, Ortenberg, Germany).

### 2.11. Glucose-Stimulated Insulin Secretion Assay (GSIS)

A comprehensive GSIS assessment was performed to evaluate the effects of metabolites derived from diet-manipulated bacteria on mammalian cells. Non-cytotoxic metabolite concentrations were added to 400,000 INS-1 832/3 cells for 4 h (24-well plates). For the insulin secretion assay (post-metabolite exposure), the cells were washed twice with 300 µL of glucose-free Krebs-Ringer Bicarbonate HEPES (KRBH) buffer (116 mM NaCl, 1.8 mM CaCl2.2(H_2_O), 0.8 mM MgSO_4_ 7(H_2_O), 5.4 mM KCl, 1 mM NaH_2_PO_4_2(H_2_O), 26 mM NaHCO_3_, 0.5% BSA, and 10 mM HEPES, pH 7.4), before pre-incubation for 1 h in 500 µL glucose-free KRBH buffer. Subsequently, the cells were incubated for 1 h with 500 µL of 2.8 mM basal glucose in KRBH buffer, followed by an additional hour with 500 µL of 16.7 mM stimulatory glucose in KRBH buffer for all metabolite-exposed samples, excluding controls (positive control was high glucose (16.7 mM). In contrast, the negative control was glucose-free KRBH from non-exposed cells). The supernatants were collected and stored at −80 °C until analysis. The insulin levels were quantified using an insulin enzyme ELISA following the manufacturer’s instructions (Mercodia, Uppsala, Sweden).

### 2.12. ERK Pathway Activation in INS-1 832/3 Cells

Western blotting was used to assess the intracellular protein levels associated with insulin signaling following exposure to metabolite extracts and glucose stimulation. The cells were lysed in ice-cold RIPA buffer supplemented with protease and phosphatase inhibitors (Thermo Fisher Scientific). The protein was quantified using the Bradford Assay and separated by SDS-PAGE before wet-transfer to PVDF membrane for 2 h at 70 V, 4 °C. Next, the membranes were incubated with primary antibodies overnight at 4 °C (anti-phospho p44/42 MAPK (ERK1/2) Thr202/Tyr204 (1:5000), anti-P44/42 MAPK (ERK1/2) (1:5000), and anti-α-tubulin (1:5000) (Abcam, Cambridge, UK)). The following day, the membranes were incubated with HRP-conjugated or infrared-conjugated secondary antibodies (Goat anti-Rabbit HRP 1:5000 (Vector Labs, Newark, CA, USA); anti-mouse 1:5000 (LICORBio, Lincoln, NE, USA) at room temperature for 90 min. Protein bands were detected using chemiluminescence and imaged via a LICORBio Odyssey system. Finally, densitometric analysis was performed using Image Studio Lite v5.2, with protein expression normalized to α-tubulin.

### 2.13. Statistical Analysis

All experiments had a minimum of three independent replicates (*n* = 3). The statistical analysis was performed by comparing with respective controls using Tukey’s and Šidák’s multiple comparisons associated with one and two-way ANOVA analysis using GraphPad Prism 10.3.1 (La Jolla, CA, USA). The data is expressed as the mean ± standard error of the mean (SEM).

## 3. Results

### 3.1. Influence of Dietary Conditions on the Growth of Bacterial Populations

**Figure 1:**
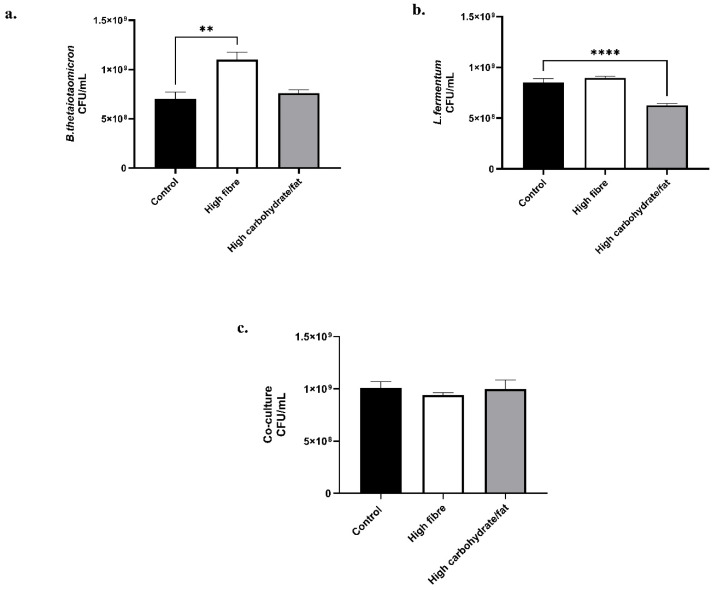
**Influence of *in vitro* dietary manipulation on gut bacterial populations.** The impact of diets on bacterial growth was assessed by measuring CFU/mL through serial dilutions. (**a**) *B. thetaiotaomicron*; (**b**) *L. fermentum* and (**c**) co-culture of *B. thetaiotaomicron* and *L. fermentum*, were incubated in FAA broth medium for 24 h at 37 °C, supplemented with either a high-fibre or a high-carbohydrate/fat diet, or a control (no diet). The values are depicted as the mean ± SEM (*n* = 3) with significance presented by ** *p* < 0.005, and **** *p* < 0.0005.

The effect of dietary variation on the growth dynamics of bacterial monocultures and co-cultures was assessed under anaerobic conditions. In high-fibre cultures, *B. thetaiotaomicron* exhibited a significant increase in growth (Figure 1a), consistent with its role as a primary fibre-degrading bacterium. In contrast, *L. fermentum*, which lacks the ability to degrade complex polysaccharides, showed only a modest increase. No significant change in growth was observed in the co-culture under these conditions.

Conversely, cultures supplemented with high-carbohydrate/high-fat diets significantly reduced the growth of *L. fermentum*, whereas *B. thetaiotaomicron* and the co-culture remained unaffected. These findings indicate that dietary composition exerts a substantial influence on microbial growth. Notably, the absence of significant changes in co-culture growth under varying dietary conditions suggests that microbial interactions—such as cross-feeding or metabolite exchange—may confer a compensatory effect.

### 3.2. Untargeted Characterisation of Extracted Metabolites

**Figure 2:**
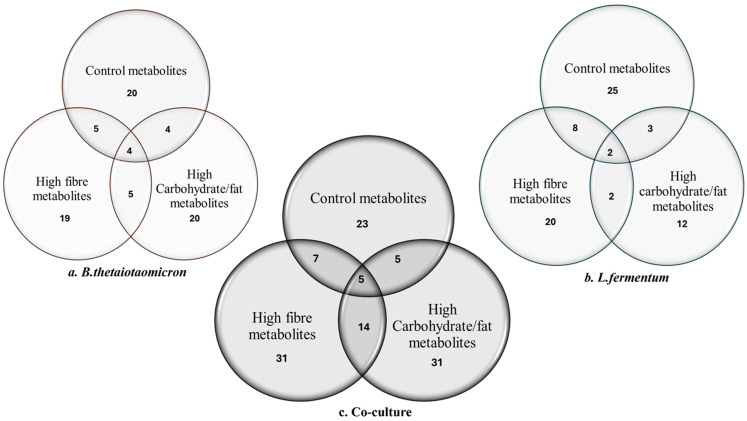
**Untargeted GC-MS analysis.** The Venn diagram illustrates the quantity of peaks eluted under various dietary conditions, including control, high fibre, and high carbohydrates/fat for (**a**) *B. thetaiotaomicron*; (**b**) *L. fermentum* and (**c**) co-culture.

Untargeted mass spectrometry of extracted metabolites revealed a diverse array of compounds associated with key metabolic pathways, including SCFA metabolism, amino acid metabolism, secondary metabolite biosynthesis, diketopiperazine formation, and fatty acid amide metabolism (Table 1). Notably, metabolite profiles varied between monocultures and co-cultures, as well as across dietary conditions (Figure 2; Table 1). For example, isovaleric acid, 3-methyl-butanoic acid, and 2-methyl-hexanoic acid were predominantly detected in control and high-fibre diets, reflecting active fermentation processes that support gut health and immune modulation [32,33].

Chromatographic analysis of samples from each culture yielded distinct peaks corresponding to individual metabolites, with detailed chromatograms and peak lists provided in Supplementary Figure S3.

### 3.3. Quantification of Bacterial Metabolites

Following the approach described by [30], selected metabolites were quantified using a PEG column, eliminating the need for derivatization. Analytical standards including acetic acid, propionic acid, butyric acid, and the internal standard 2-ethylbutyric acid were reconstituted in hexane and analyzed in full-scan mode across an *m*/*z* range of 20–500 amu to confirm target *m*/*z* ratios and retention times (Table 2).

The methodology described above was validated by determining the calibration ranges of SCFAs using an internal standard (2-ethylbutyric acid). The limit of detection (LOD) and limit of quantification (LOQ) are listed in Table 3.

**Figure 3:**
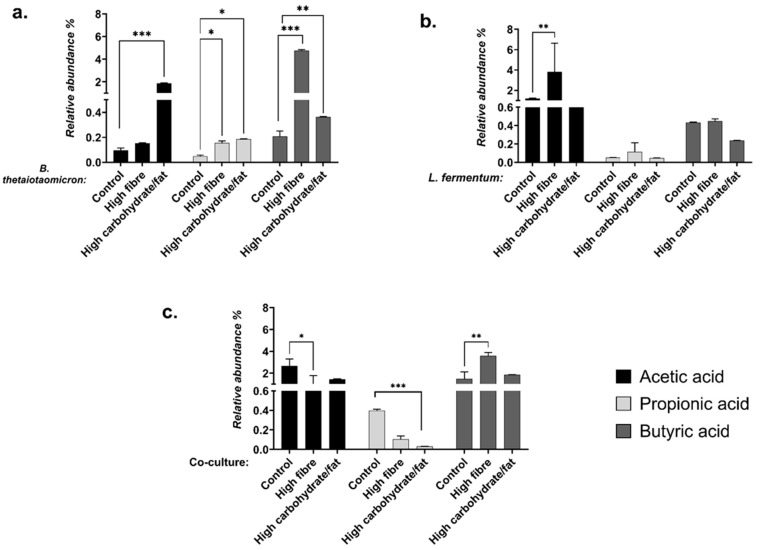
Relative abundance (%) of SCFAs (acetic acid, propionic acid, and butyric acid) in dietary manipulation metabolites quantified by GC-MS. The data was calculated based on the response ratio of each SCFA peak area to the internal standard (**a**) *B. thetaiotaomicron*; (**b**) *L. fermentum* and (**c**) Co-culture. The data is expressed as the percentage of each SCFA relative to the total SCFA content. The values are depicted as the mean ± SEM (*n* = 3) with significance presented by * *p* < 0.05, ** *p* < 0.005 and *** *p* < 0.0005, respectively.

**Figure 4:**
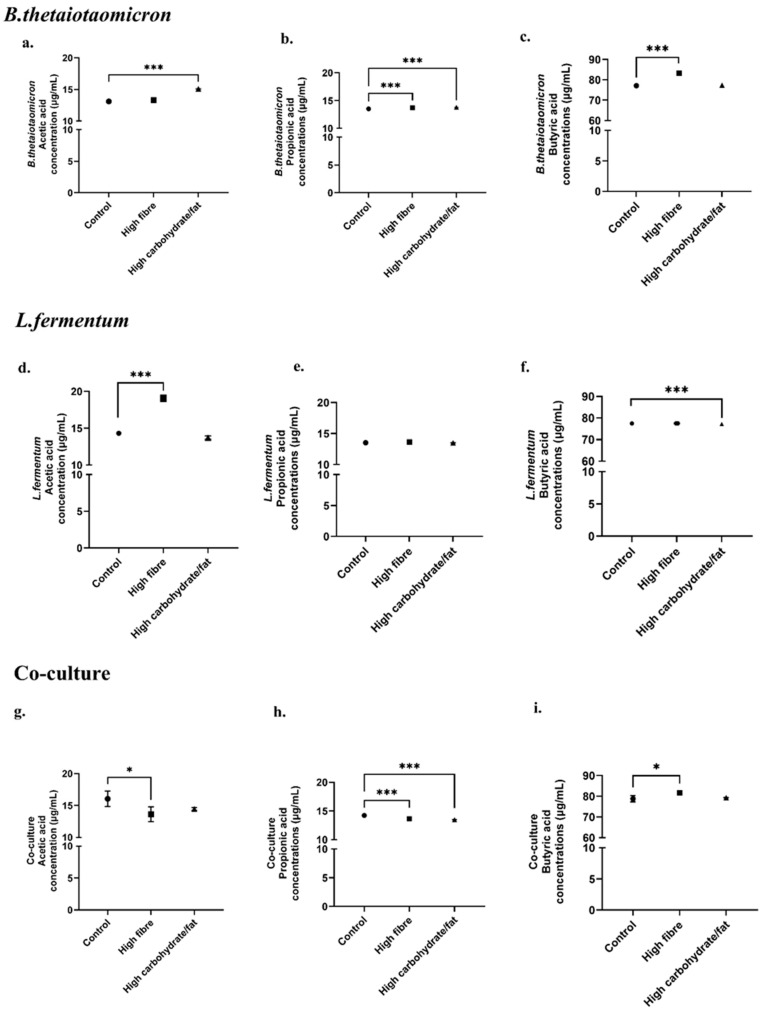
**The concentrations of SCFAs.** *B. thetaiotaomicron* (**a**–**c**); *L. fermentum* (**d**–**f**) and co-culture (**g**–**i**) measured using standard curves derived from the linearity of each specific fatty acid (*n* = 3). The values are depicted as the mean ± SEM (*n* = 3) with significance presented by * *p* < 0.05 and *** *p* < 0.0005, respectively. Circle—control (no diet metabolites); square—high fibre metabolites, triangle—high carbohydrate/fat metabolites.

SCFA concentrations in metabolites extracted from monocultures and co-cultures were determined using selected ion monitoring (SIM). These data provided insights into the relative abundance (%) of individual SCFAs compared to other metabolites, enabling identification of dominant analytes (Figure 3). Overall SCFA concentrations were quantified across dietary conditions (Figure 3 and Figure 4).

In *B. thetaiotaomicron*, acetic acid levels were significantly elevated under high-carbohydrate/high-fat conditions compared to control (*p* = 0.0001; Figure 4a). Propionic acid increased under both high-fibre (*p* = 0.0008) and high-carbohydrate/high-fat diets (*p* = 0.0002; Figure 4b), whereas butyric acid was most abundant under high-fibre conditions (*p* < 0.0001; Figure 4c).

For *L. fermentum*, acetic acid was significantly higher in high-fibre cultures compared to control (*p* < 0.0001; Figure 4d), while propionic acid remained low across all conditions (Figure 4e). Butyric acid decreased significantly under high-carbohydrate/high-fat conditions (*p* < 0.0001; Figure 4f).

Finally, in co-culture, acetic and propionic acid were significantly elevated under control conditions (*p* = 0.0429) compared to high-fibre (*p* = 0.0001) and high-carbohydrate/high-fat diets (*p* < 0.0001; Figure 4g,h). Conversely, butyric acid was significantly higher under high-fibre conditions (*p* = 0.0198; Figure 4i).

### 3.4. Endotoxin Removal from a Subset of Extracted Metabolites

Endotoxin removal from a subset of extracted metabolites was essential prior to assessing biological effects in mammalian cells, particularly given the presence of Gram-negative *B. thetaiotaomicron* [47]. This step ensured that observed cellular responses could be attributed to metabolites rather than lipopolysaccharides (LPS), or a combination of both. Consistent with expectations, endotoxin concentrations were minimal in *L. fermentum* (<0.01 EU/mL) compared to *B. thetaiotaomicron*.

### 3.5. Impact of Diet-Manipulated Metabolites on INS-1 832/3 Cell Viability

The potential cytotoxicity of bacterial metabolites was evaluated at 24 and 48 h. The data revealed a significant, albeit modest, cytotoxic effect at high concentrations (1000 µg/mL) of *B. thetaiotaomicron* metabolites derived from control and carbohydrate/fat-supplemented diets, as well as *L. fermentum* metabolites from control and high-carbohydrate/high-fat conditions (Supplementary Figure S2). Notably, all subsequent experiments were conducted using metabolite concentrations confirmed to be non-cytotoxic.

### 3.6. Insulin Release and Intracellular ERK1/2 Signaling Induced by the Diet-Manipulated Bacterial Metabolites

**Figure 5:**
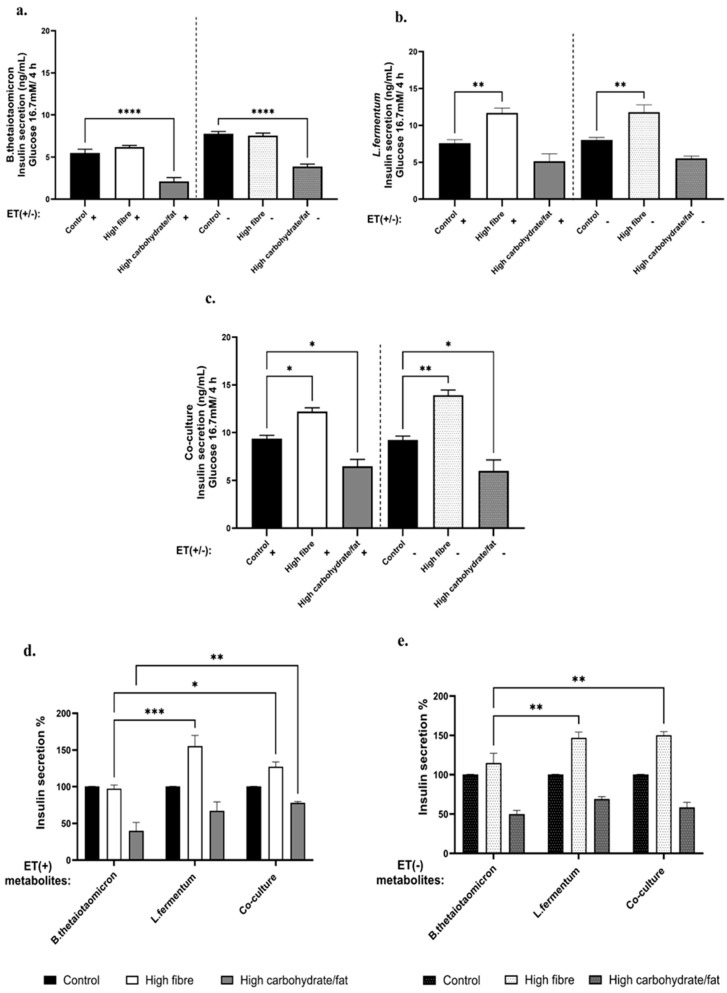
**The GSIS assay was utilized to quantify insulin secretion from INS-1 832/3 cells.** The cells were exposed to dietary metabolites (500 μg/mL, ET+/ET−) derived from monocultures and co-cultures for a duration of 4 h. (**a**) *B. thetaiotaomicron*; (**b**) *L. fermentum*; (**c**) Co-culture; (**d**) dietary influenced insulin secretion expressed as % as compared to control before LPS removal and (**e**) dietary influenced insulin secretion expressed as % as compared to control before LPS removal. The values are depicted as the mean ± SEM (*n* = 3) with significance presented by * *p* < 0.05, ** *p* < 0.005, *** *p* < 0.0005) and **** *p* < 0.0001).

GSIS was performed following exposure of INS-1 832/3 cells to diet-modulated bacterial metabolites at non-cytotoxic concentrations. Metabolites derived from high-carbohydrate/high-fat diets originating from both monocultures and co-culture (Figure 5a–c) significantly impaired insulin secretion across all experimental groups (ET+/–), indicating a detrimental effect on insulin sensitivity. In contrast, metabolites from high-fibre diets, particularly those from *L. fermentum* and co-culture (Figure 5b,c), enhanced insulin secretion, suggesting a protective role of fibre-associated metabolites. *B. thetaiotaomicron* (Figure 5a) exhibited only modest changes under high-fibre conditions, which were not statistically significant.

Notably, comparisons before and after LPS removal (Figure 5d,e) revealed that metabolites from high-fibre and high-carbohydrate/high-fat diets produced by *B. thetaiotaomicron* induced significantly lower insulin secretion compared to those from *L. fermentum* and co-culture, highlighting the combined influence of LPS and bacterial metabolites. However, insulin secretion remained lower in high-carbohydrate/high-fat conditions even after LPS removal, indicating that diet-derived metabolites alone exert a detrimental effect.

Insulin secretion from pancreatic β-cells in response to high glucose is tightly coupled with insulin gene expression, ensuring β-cell function and glucose homeostasis. ERK1/2 mitogen-activated protein kinases play a pivotal role in this process [48,49]. Previous studies have demonstrated that ERK1/2 activity is required for glucose-stimulated insulin gene expression via phosphorylation and activation of transcription factors such as Beta2/NeuroD1 and PDX-1, leading to enhanced insulin promoter activity [50]. Accordingly, ERK1/2 expression and phosphorylation were assessed in INS-1 832/3 cells following GSIS to evaluate β-cell responses to bacterial metabolites.

**Figure 6:**
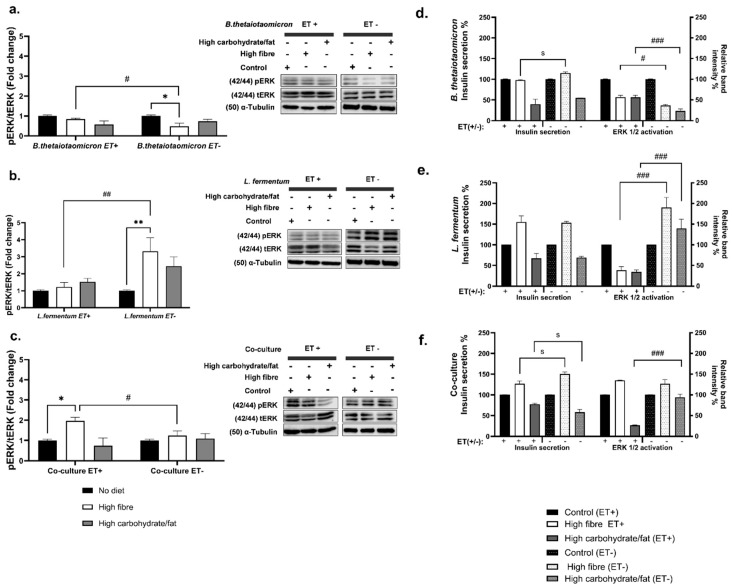
**Effects of bacterial metabolite-induced ERK1/2 activation in INS-1 832/3 cells.** The cells were treated with 500 μg/mL of dietary bacterial metabolite extracts (ET+/−) from (**a**) *B. thetaiotaomicron*; (**b**) *L. fermentum*; and (**c**) co-culture for 4 h before GSIS. The relative band intensity was calculated as the ratio of phospho-ERK1/2 to total ERK1/2. The comparison of insulin secretion after GSIS alongside expression of ERK1/2 phosphorylation under the influence of ET+/− dietary metabolites of (**d**) *B. thetaiotaomicron*; (**e**) *L. fermentum*, and (**f**) co-culture, in which the data is expressed relative to the response in the control condition, set as 100%. Insulin secretion percentages were measured in parallel. Significance * indicates the comparison between respective control and diets in ERK1/2 activation, s indicates the comparison between insulin secretion between ET+ and ET−, while # denotes the comparison between individual ET+ and ET− in ERK1/2 activation. The data is presented as mean ± SEM, with representative blots shown alongside quantification (*n* = 5; */s/# *p* < 0.05, **/## *p* < 0.005, and ### *p* < 0.0005).

For *B. thetaiotaomicron* (Gram-negative), ERK1/2 phosphorylation was significantly higher in samples without LPS removal (ET+) (*p* = 0.0260) (Figure 6a), suggesting that LPS enhances ERK1/2 activation, likely via inflammatory signaling [14,51]. Under high-fibre conditions, ERK1/2 phosphorylation increased in ET+ (*p* = 0.014), while insulin secretion was modestly higher in ET− samples (*p* = 0.0493) despite reduced ERK1/2 activation in ET− (Figure 6d). In contrast, high-carbohydrate/high-fat metabolites induced greater ERK1/2 phosphorylation in ET+ samples compared to ET− samples (*p* < 0.0001), although insulin secretion decreased in both conditions.

For *L. fermentum*, LPS presence significantly reduced ERK1/2 phosphorylation compared to ET− samples (*p* = 0.0037) (Figure 6b). High-fibre metabolites promoted insulin secretion in ET+ samples despite lower ERK1/2 activation (*p* < 0.0001), whereas ET−samples exhibited increased ERK1/2 activation with high insulin secretion. Conversely, high-carbohydrate/high-fat metabolites caused reduced ERK1/2 phosphorylation (*p* < 0.0001) and insulin secretion in ET+ samples, while ET− samples showed moderate ERK1/2 activation and elevated insulin secretion (Figure 6e).

In the co-culture, high-fibre metabolites induced ERK1/2 activation in both ET+ increased compared to ET− samples (*p* = 0.0411) (Figure 6c), but insulin secretion was greater in ET− samples (*p* < 0.05). Conversely, high-carbohydrate/high-fat metabolites without LPS removal resulted in markedly reduced ERK1/2 activation (*p* < 0.0001), with insulin secretion remaining moderately low in both conditions (Figure 6f).

## 4. Discussion

This study employed a novel *in vitro* approach to examine interactions between dietary composition, gut bacterial growth, metabolite profiles, and downstream effects on metabolic pathways relevant to T2DM. The data demonstrated a clear cause-and-effect relationship between diet and bacterial growth, associated shifts in metabolite abundance, and their influence on disease progression.

To mimic eubiotic gut conditions, low-viscosity, low-degree-of-polymerization carboxymethyl cellulose (CMC) was selected as the high-fibre substrate. CMC is a chemically defined, water-soluble polysaccharide composed of β-D-glucose and β-D-glucopyranose units substituted with carboxymethyl groups and linked via β-1,4-glycosidic bonds [52,53]. Its solubility and structural uniformity make it an ideal substrate for controlled *in vitro* studies [54,55,56,57].

Unlike resistant starches or oligosaccharides, CMC can be efficiently utilized by fibre-degrading bacteria such as *B. thetaiotaomicron*, a Gram-negative obligate anaerobe specialized in complex carbohydrate digestion. This species encodes glycoside hydrolases and polysaccharide utilization loci (PULs) that enable the breakdown of polysaccharides and fermentation into short-chain fatty acids (SCFAs), including acetate, propionate, and butyrate. Consistent with this, *B. thetaiotaomicron* exhibited significant growth under high-fibre conditions in this study (Figure 1a). These findings align with previous reports demonstrating cellulose-degrading activity in CMC media by *Bacteroides* spp., linked to PULs encoding glycoside hydrolases, β-1,4-glucanases, carbohydrate esterases, and related enzymes [58,59,60,61,62,63].

*L. fermentum*, a Gram-positive facultative anaerobe, exhibited modest growth under high-fibre conditions. This species primarily metabolizes simple sugars via glycolysis and the phosphoketolase pathway [64,65,66]. CMC was selected as the fibre source because it is not degraded by human enzymes, including cellulases that cleave β-1,4-glycosidic bonds in cellulose and its derivatives [60,66]. Consequently, CMC passes through the gastrointestinal tract without absorption or metabolism by host cells, remaining available for bacterial fermentation. This property makes CMC an ideal substrate for controlled *in vitro* studies of microbial metabolism and SCFA production.

To simulate dysbiotic dietary conditions, a combination of high glucose and low-density lipoprotein (LDL) was used. Elevated glucose levels are known to increase intestinal permeability, promote inflammation, and disrupt SCFA balance. LDL provides a host-like lipid source for gut bacteria *in vitro*, mimicking high-fat dietary conditions in the absence of host digestion. Normally, dietary fats are absorbed in the small intestine, leaving minimal lipids for colonic microbes [67]. However, during high-fat intake or inflammation, LDL components such as cholesterol and phospholipids can reach the colon. Certain gut bacteria, including *Lactobacillus* and *Bacteroides* spp., produce lipases and bile salt hydrolases (BSH), enabling lipid breakdown [67,68,69,70].

Ref. [71] demonstrated that gut bacteria metabolize endogenous cholesterol synthesized by the liver. Their study identified sterol metabolism A genes in bacterial species from the *Bacillota* and *Bacteroidota* phyla, including *Bacteroides dorei* and *Lactobacillus*. These genes enable the conversion of cholesterol into coprostanol, reducing intestinal cholesterol absorption and lowering circulating LDL levels. Consistent with these findings, Ref. [12] employed an *in vivo* mouse model using cholesterol tagged with an alkyne group and a novel labelling technique. Their results revealed that *Bacteroides* species, particularly *B. thetaiotaomicron*, actively metabolize cholesterol by converting it into cholesterol-3-sulfate via the enzyme BT_0416. This provides direct evidence that gut bacteria participate in cholesterol metabolism. Accordingly, LDL serves as a relevant *in vitro* lipid source for studying microbial responses under high-fat or dysregulated conditions. In our study the data shows that high-glucose and LDL conditions impair the metabolic functions of *L. fermentum* (Figure 1b). We hypothesize that combined exposure to glucose and LDL overwhelms bacterial metabolic pathways, promoting dysbiosis. The literature suggests that such dysbiotic states activate inflammatory pathways, potentially via TLR4 signaling and endotoxemia, contributing to insulin resistance and the progression of T2DM [7,72,73].

In contrast, co-culture conditions showed no significant changes in bacterial growth dynamics across diets (Figure 1c). This stability likely reflects synergistic interactions between species, facilitated by cross-feeding and metabolic flexibility involving glycolysis, fatty acid metabolism, and fermentation processes [8,9]. These mechanisms support SCFA production and help maintain a stable gut environment despite dietary perturbations. Future studies with refined dietary manipulations will further elucidate these interactions.

Next, to characterize metabolic shifts, metabolites were extracted using an optimized protocol [26]. Untargeted analysis revealed distinct signatures in high-fibre diets, including short- and branched-chain fatty acids (Table 2). In contrast, high-carbohydrate/high-fat diets lacked several previously identified metabolites, indicating that unhealthy diets profoundly alter microbial metabolism.

In this study, untargeted GC-MS analysis revealed long-chain and branched aromatic compounds unique to high-carbohydrate/high-fat diet groups in both mono- and co-cultures. These included L-leucine, *N*-cyclopropyl carbonyl-butyl ester, cyclo(L-prolyl-L-valine), cyclohexane, and carboxylic acid 1-tert-butyl (Table 1). Previous studies suggest that elevated levels of these metabolites, commonly associated with high-carbohydrate/high-fat diets, may contribute to modulation of metabolic disease. Additionally, signatures of secondary metabolite synthesis, altered bile acid metabolism, lipid degradation products (e.g., alkanes), and pathways linked to fatty acid amide metabolism were observed (Supplementary Tables S1–S3). These findings indicate a microbiota-driven metabolic shift that may underlie impaired insulin sensitivity [74,75,76].

Diketopiperazines, including cyclo(L-prolyl-L-valine) and cyclo(leucylprolyl), were detected in high-fibre and control groups, reflecting active microbial fermentation of branched-chain amino acids (BCAAs) using peptones and yeast extract as nitrogen sources in FAA broth. Previously [77] reported that cyclo(leucylprolyl), identified in bacterial conditioned media from *Pseudomonas aeruginosa* and *Aeromonas dhakensis*, exhibited strong cytotoxicity against HeLa cells (89.5% inhibition) and moderate toxicity against normal keratinocytes (59% inhibition). Thus, detection of cyclo(leucylprolyl) in high-fibre conditions suggests a potential protective role of fibre-rich diets in disease modulation. Furthermore, phenolic compounds such as phenol and 2,2′-methylenebis, known antioxidants, were identified, underscoring the complex metabolic processes of gut bacteria and their influence on host health.

The metabolite 1,3-propanediol plays a crucial role in gut microbial central metabolism, primarily through glycerol fermentation. *Lactobacillus* spp. utilize a B12-dependent glycerol/diol dehydratase to convert glycerol into 3-hydroxypropionaldehyde, which is subsequently reduced to 1,3-propanediol and then to propanol, an intermediate in propionate formation [36,37,78]. This process helps regenerate NAD^+^ and maintain redox balance in *Lactobacillus* spp. and other members of the *Bacillota* phylum.

The detection of this metabolite exclusively in co-cultures underscores the importance of cross-feeding between species [7,26]. Pathways involving *L. fermentum* and *B. thetaiotaomicron* illustrate a synergistic microbial strategy that enhances energy efficiency and fermentation, leading to improved metabolite production. In a healthy diet, these interactions support beneficial fermentation and metabolic health. Conversely, in high-carbohydrate or high-fat diets, excessive glycerol fermentation can disrupt microbial balance and generate harmful by-products such as acrolein from 3-HPA, which may exacerbate oxidative stress and inflammation [37,79].

**Figure 7:**
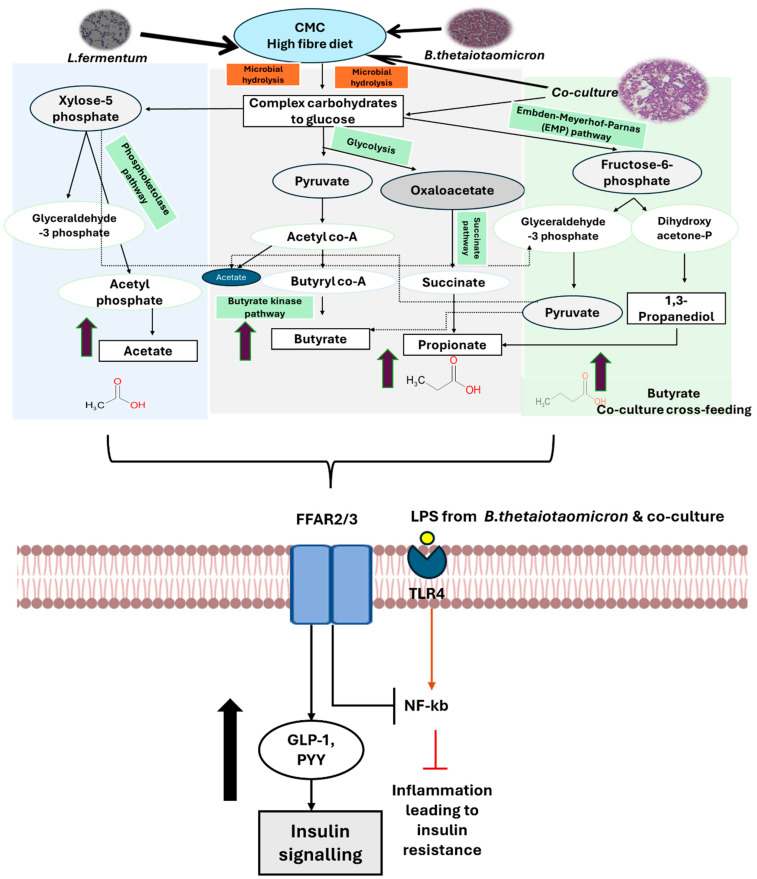
**The proposed biochemical pathways involved in synthesizing and regulating SCFAs under a high fibre diet.** In *B. thetaiotaomicron*, a Gram-negative bacterium that degrades fibre, SCFA synthesis occurs following glycolysis of CMC, resulting in elevated levels of butyrate and propionate via the butyrate kinase and succinate pathways. The LPS produced by this bacterium are counteracted by butyrate and propionate, potentially leading to reduced inflammation via TLR4 and improved insulin sensitivity. On the other hand, *L. fermentum*, a probiotic Gram-positive bacterium that breaks down CMC via the phosphoketolase pathway, leading to a higher production of acetate compared to other SCFAs, with no LPS generated. However, in the co-culture, the production of 1,3-propanediol metabolite was identified along with synergistic metabolic pathways that enhance the production of butyrate, propionate, and acetate. This highlights the cross-feeding mechanisms, indicated by dotted lines, and demonstrates the buffering effects of the co-culture in response to LPS-regulated inflammation, leading to enhanced insulin sensitivity. The broad brown arrows with green border indicate the increase of SCFAs under CMC diet, whereas the broad black arrow indicates the possible downstream effect of these metabolites on INS-1 832/3 cells leading to increased insulin signaling. This figure is adapted from Guraka et al., 2024 and 2025 [7,26].

**Figure 8:**
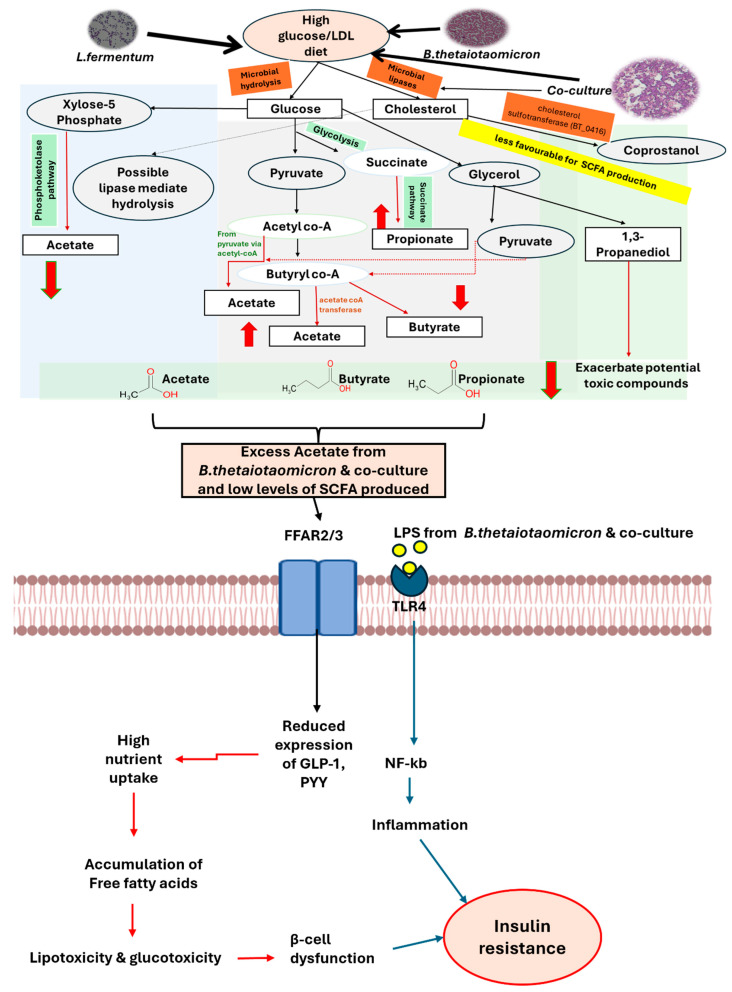
**The proposed biochemical pathways involved in the synthesis and regulation of SCFAs in a high carbohydrate/fat diet.** In *B. thetaiotaomicron*, a Gram-negative bacterium that degrades glucose, the process following glycolysis produces excess acetate via the acetyl-CoA and butyryl-CoA pathways through the acetyl-CoA transferase route. This bacterium shows lower levels of propionate and butyrate, coupled with high fat (LDL) through the microbial lipases activity, potentially by *B. thetaiotaomicron*, resulting in the production of coprostanol via cholesterol sulfotransferase by degrading free cholesterol from LDL. This provides less favorable conditions for SCFA production. Alongside the LPS produced by this bacterium, increased TLR4 activation leads to inflammation and insulin resistance. In contrast, the other monoculture, *L. fermentum*, with its heterofermentative nature, breaks down high glucose via the phosphoketolase pathway, resulting in lower acetate production and displaying glucotoxic effects, with no LPS generated. It also facilitates lipase-mediated hydrolysis of LDL, releasing free fatty acids. However, in their co-culture, SCFA production is reduced, potentially due to the accumulation of toxic intermediate metabolites, such as 1,3-propanediol and free fatty acids. The highlighted dysbiotic cross-feeding mechanisms are indicated by dotted lines, demonstrating the combined effects of gluco- and lipotoxicity and LPS-induced inflammation, potentially leading to insulin resistance. The thin black/red/blue arrows show the direction and change in the end product in the pathways, and downstream signalling; Broad red arrows with green borderindicates the significant effect in decrease of SCFAs under high glucose/LDL; whereas the broad red arrowindicates the increase and decrease of imbalanced SCFA production. These figures are adapted from Guraka et al., 2024 and 2025 [7,26].

Following targeted characterization, several key short-chain fatty acids (SCFAs) produced by the bacteria were quantified after dietary manipulation. The proposed biochemical pathways involving these metabolites, their association with dietary changes, and their influence on β-cell function are illustrated in Figure 7 and Figure 8.

Under high-fibre conditions, butyric and propionic acid levels increased in *B. thetaiotaomicron* (Figure 3a and Figure 4c). Butyrate production is primarily mediated by species within the *Bacillota* phylum [1,80,81]. Data from the Integrated Microbial Genomes and Microbiomes database confirm that the genome of *B. thetaiotaomicron* encodes enzymes essential for butyrate synthesis, notably butyrate kinase (EC 2.7.2.7), and for propionate production, such as acetate kinase (EC 2.7.2.1) [82]. These findings indicate that *B. thetaiotaomicron* efficiently converts dietary fibres into butyrate and propionate.

In contrast, fibre-rich diets for *L. fermentum* (Figure 4d) resulted in higher acetic acid concentrations, consistent with its heterofermentative metabolism (Figure 7). *L. fermentum* lacks the gene encoding phosphofructokinase, a key glycolytic enzyme, leading to the production of lactate, acetate, ethanol, and CO_2_ [83]. Fermentable vegetable fibres have been shown to promote rapid acetic acid accumulation by *L. fermentum*, highlighting its role in shaping the SCFA profile and influencing gut microbial ecology and host metabolism [84].

The importance of co-culture becomes evident in the enhanced butyric acid production under high-fibre conditions (Figure 3c and Figure 4i). Butyrate serves as a major energy source for colonocytes and exhibits anti-inflammatory and anti-carcinogenic properties [1,8,24,26]. The increased SCFA levels in co-culture suggest synergistic metabolic interactions between *B. thetaiotaomicron* and *L. fermentum*, likely through cross-feeding mechanisms (Figure 7). This cooperation underscores the ecological and functional relevance of co-cultures in replicating the complex microbial environment of the gut [7].

A high-carbohydrate, high-fat diet significantly increased acetic acid levels in *B. thetaiotaomicron* (Figure 3a and Figure 4a), while markedly reducing acetic, propionic, and butyric acid concentrations in *L. fermentum* and the co-culture (Figure 4d–i). Under physiological conditions, acetate plays a role in regulating central metabolism. However, excessive acetate, particularly in high-fat diets, can stimulate lipogenesis, contributing to obesity and insulin resistance [84,85] (Figure 8). This is supported by recent findings showing that elevated acetate levels drive lipogenesis and fat storage via insulin and ghrelin signaling in rats fed a high-fat diet [86]. Consequently, dysbiotic diets may amplify toxic intermediates produced by lipid pathways, such as those involved in triacylglycerol synthesis. This creates a vicious cycle of glucotoxicity, lipotoxicity, and inflammation, ultimately impairing β-cell function and promoting insulin resistance and metabolic dysfunction.

GSIS data revealed that metabolites extracted from bacteria exposed to a high-carbohydrate, high-fat diet induced a dysbiotic metabolic shift. This shift resulted in imbalanced SCFA levels and the presence of numerous long-chain secondary aromatic compounds, which impaired insulin secretion regardless of LPS presence. These effects were most pronounced in *B. thetaiotaomicron* and the co-culture (Figure 5a,c).

The study demonstrated that metabolites from dysbiotic conditions negatively affect β-cell function, characterized by reduced butyrate (in *L. fermentum*) and propionate (in co-culture), alongside elevated acetate and propionate (in *B. thetaiotaomicron*). This suggests that low or unbalanced SCFA levels driven by high glucose and fat may reduce FFAR2/3 activation, thereby disrupting insulin secretion from pancreatic β-cells and impairing glucose metabolism.

In contrast, GSIS data indicated that high-fibre diets significantly enhanced insulin secretion. In monoculture, SCFAs derived from high-fibre conditions, particularly acetate from *L. fermentum*, exhibited protective effects by promoting insulin release (Figure 5b). Meanwhile, *B. thetaiotaomicron* remained stable under ET+/− conditions (Figure 5a). These findings imply that the insulinotropic effects of SCFAs (propionate and butyrate), which regulate glucose homeostasis, are mediated via FFAR3-Gαi/o pathways associated with high-fibre diets. Notably, co-culture conditions (Figure 5c) showed a greater increase in insulin secretion than monocultures, particularly involving *B. thetaiotaomicron*. This enhancement likely reflects cross-feeding interactions between *B. thetaiotaomicron* and *L. fermentum*, creating favourable conditions for insulin production regardless of LPS presence.

Glucose-dependent activation of ERK1/2 is central to the regulation of GSIS in β-cells. This response is mediated by membrane depolarisation, calcium influx, and subsequent engagement of the MAPK pathway, culminating in ERK1/2 activation, a key signalling axis for insulin release [49,87,88]. To determine whether diet-induced changes in insulin secretion align with ERK1/2 signaling, ERK1/2 phosphorylation was assessed alongside insulin release, providing mechanistic insight into β-cell regulation under distinct metabolic conditions. Overall, insulin signaling and ERK1/2 activation varied with bacterial species and dietary manipulation.

In *B. thetaiotaomicron*, metabolites derived from a high-fibre diet in the presence of endotoxins were associated with decreased insulin signaling, supported by elevated ERK1/2 activation when LPS was not removed (Figure 6a). By contrast, under ET− conditions (following LPS removal), ERK1/2 activation was reduced, and insulin secretion increased. These findings indicate that LPS can attenuate the protective metabolic effects of a high-fibre diet by augmenting ERK1/2 activity (noting that LPS removal is an experimental intervention). Consistent with this, removal of LPS was associated with diminished ERK1/2 activity and enhanced insulin release, suggesting that LPS perturbs metabolic homeostasis via ERK1/2-dependent and additional pathways. Thus, for *B. thetaiotaomicron*, LPS appears to be a major contributor to the observed biological effects (Figure 4b,c), in line with its recognized role in the pathophysiology of type 2 diabetes [49,50,87,88]. Conversely, metabolites from a high-carbohydrate/high-fat diet under ET− conditions showed substantially reduced ERK1/2 phosphorylation accompanied by decreased insulin secretion (Figure 6a). These observations suggest that insulin secretion is modulated not only by the presence of LPS and ERK1/2 activation, but also, critically, by dietary composition. This conclusion is supported by reduced butyric acid (Figure 4c), elevated acetic and propionic acid (Figure 4a), and the presence of complex aromatic long-chain compounds (Table 1), all of which are implicated in disease progression.

In contrast, *L. fermentum* under a high-fibre diet efficiently produced short-chain fatty acids (SCFAs), particularly acetic acid (Figure 4d), and sustained higher insulin levels irrespective of LPS removal, consistent with its Gram-positive status. Under high-carbohydrate/high-fat conditions, SCFA levels were lower, especially butyric acid (Figure 4f), and this was associated with markedly reduced ERK1/2 activation and decreased insulin secretion (Figure 6b).

In high-fibre co-culture conditions, insulin secretion and ERK1/2 activation were increased regardless of LPS removal (Figure 6c). This suggests that metabolites—particularly butyric acid—may mitigate the inhibitory effects of LPS and enhance β-cell functionality. The observed upregulation of ERK1/2 signaling could reflect a compensatory or stimulatory mechanism whereby dietary metabolites, including butyric acid and other bioactive compounds, activate intracellular pathways beyond the classical FFAR3 Gαi/o inhibitory axis that regulates insulin secretion. These findings highlight how interspecies bacterial interactions can amplify beneficial metabolic effects by synergistically modulating metabolite production and ERK1/2 activation.

Under high-carbohydrate/high-fat diet conditions, reduced ERK1/2 activation was associated with lower insulin secretion. This supports the concept that diminished SCFA levels, particularly propionic acid, in the context of glucotoxicity and lipotoxicity contribute to impaired β-cell function. Under physiological conditions, propionate derived from the diet enhances glucose-stimulated insulin release via FFAR2 activation and downstream pathways, such as protein kinase C [89], while also protecting β-cells from apoptosis induced by free fatty acids and inflammatory cytokines. Collectively, the data indicate that a dysbiotic diet alters gut microbiota composition, reducing SCFA production (propionate, acetate, and butyrate; Figure 8). This decline compromises protective signaling mechanisms, promotes β-cell dysfunction, and ultimately impairs insulin secretion.

## 5. Conclusions

This *in vitro* study highlights the complex interplay among gut bacteria, dietary composition, bacterial metabolites, and their biological effects, illustrating a link in the cause-and-effect dynamics between the gut microbiota and the pathogenesis of T2DM. The findings demonstrate that a high-fibre diet improves glucose metabolism by activating ERK1/2 in pancreatic β-cells, driven by increased butyric acid and other SCFAs such as acetic and propionic acids, which act synergistically in co-culture regardless of LPS influence. In contrast, high-carbohydrate/high-fat diets were associated with reduced propionic and butyric acids, elevated acetic acid, and LPS-induced inflammation, collectively diminishing ERK1/2 activation and contributing to insulin resistance.

While this study introduces a novel methodology for assessing the relationship between diet, gut microbiota, and metabolic health, it is important to acknowledge that the human gut microbiome is highly complex and extends beyond the two dominant bacterial populations examined here. Future work will incorporate a broader range of intestinal bacterial genera to better capture this complexity. Additional studies will explore other microbial metabolites, such as bile acids and secondary SCFAs, as well as the secretome under varying dietary conditions. Furthermore, we intend to refine and subdivide dietary conditions to (a) investigate the impact of long-term, low-dose metabolite exposure on T2DM; (b) better mimic diverse human diets in gut bacterial populations; and (c) determine whether complex fibres such as inulin, galacto-oligosaccharides (GOS), or fructo-oligosaccharides (FOS) influence regulatory mechanisms, including imidazole propionate synthesis, which has been linked to insulin resistance [90,91,92,93]. This level of experimental control is achievable only through an *in vitro* approach, enabling a deeper understanding of the mechanisms by which the gut microbiome influences metabolic disease pathogenesis and potentially guiding the development of therapeutic strategies based on microbial metabolites to restore eubiosis and improve metabolic outcomes.

## Figures and Tables

**Table 1:** The list of metabolites identified in the mono and co-culture of *B. thetaiotaomicron* and *L. fermentum*, and the potential gut metabolic pathways.

Potential Common Metabolites Eluted	*B. thetaiotaomicron*			*L. fermentum*			Co-Culture			Possible Pathways Linked to Gut Bacterial Metabolism
	Control	High Fibre	High Carbohydrate/ Fat	Control	High Fibre	High Carbohydrate/ Fat	Control	High Fibre	High Carbohydrate/ Fat	
Isovaleric acid; butanoic acid, 3-methyl-	✓	✓	-	-	-	-	✓	-	-	SCFA metabolism is derived from amino acid catabolism [34,35]
Hexanoic acid, 2-methyl-	✓	✓	-	✓	✓	-	✓	-	-	SCFA metabolism [34]
Glycerin	-	-	-	-	-	-	✓	-	-	Central metabolism [36,37]
L-lysine	-	-	-	✓	-	✓	-	✓	-	Cadeverine bioamine catabolism [38,39]
*N*-methylene-2-phenylethanamine	-	✓	-	-	-	-	✓	✓	-	Amino acid metabolism and secondary metabolite biosynthesis [35]
1-pentanone,1-(4-methylphenyl)-	-	-	-	-	-	-		✓	✓	Phenolic compound metabolism and secondary metabolite biosynthesis [40]
Heptanoic acid ethyl ester	-	-	-	-	-	-	✓	-	✓	Carboxylic acid from amino acid catabolism [34,35]
Cyclohexane carboxylic acid, 1-tert-butyl-	-	-	-	✓	-	-	-	-	✓	Carboxylic acid from amino acid catabolism [34,35]
1,3-propanediol	-	-	-	-	-	-	-	✓	✓	Central metabolism [36,37]
Cyclo (L, prolyl-l-valine)	✓	-	-	✓	✓	-	✓	-	✓	Diketopiperazine metabolism and peptide biosynthesis [41,42,43]
L-leucine, *N*-cyclopropyl carbonyl-butyl ester	-	-	-	-	-	-	-	✓	✓	Branched -chain amino acid catabolism [43]
Cyclo (leucycloprolyl)	✓	-	-	-	✓	-	✓	-	-	Diketopiperazine metabolism and peptide biosynthesis [41,42]
Hexadecanamide	✓	✓	✓	✓	✓	-	✓	✓	-	Fatty acid amide metabolism [44,45,46]
9-Octadecenamide, (Z)- or oleamide	✓	✓	✓	-	-	-	✓	✓	-	Fatty acid amide metabolism [44,45,46]
Nonanamide	-	-	-	-	-	-	-	✓	✓	Fatty acid amide metabolism [44,45,46]
Phenol, 2,2′-methylenebis(6-(1,1-dimethylethyl)-4-methyl-	✓	✓	✓	✓	✓	✓	✓	✓	-	Phenolic compound metabolism and secondary metabolite biosynthesis [40]

**Table 2:** Confirmative ion *m*/*z* range and retention time of targeted analytes.

Standards	Target Ion *m*/*z*	Confirmative Ions *m*/*z* (*n* = 3)	Retention Time
Acetic acid	60	43, 45	8.110
Propionic acid	74	74, 75	8.591
Butyric acid	88	60	9.061
2-ethyl butyric acid	116	88	9.692

**Table 3:** The data depicts the calibration range, limit of detection, and limit of quantification.

SCFA Standards	Calibration Range	Linear Regression (R^2^) (*n* = 3)	LOD (µg/mL, *n* = 7)	LOQ (µg/mL, *n* = 7)
Acetic acid	100–1000 µg/mL	Y = 0.008537x − 0.1101 R^2^ = 0.9940	1.41	4.26
Propionic acid	100–1000 µg/mL	Y = 0.004861x − 0.06520 R^2^ = 0.9955	1.91	5.78
Butyric acid	100–1000 µg/mL	Y = 0.007491x − 0.5757 R^2^ = 0.9906	16.1	48.5

## Data Availability

The original contributions presented in this study are included in the article/Supplementary Materials. Further inquiries can be directed to the corresponding author.

## Supplementary Materials

The following supporting information can be downloaded at https://www.mdpi.com/article/10.3390/nu18020279/s1, Table S1: Integrated peak list of *B. thetaiotaomicron* common metabolites eluted at similar retention times under control, high-fibre and high carbohydrate/fat conditions. Table S2: Integrated peak list of *L. fermentum* common metabolites eluted at similar retention times under control, high-fibre and high carbohydrate/fat conditions. Table S3: Integrated peak list of co-culture common metabolites eluted at similar retention times under control, high-fibre and high carbohydrate/fat conditions. Figure S1: (a) Bacterial identification via Sanger sequencing and BLASTn analysis revealed *Bacteroides thetaiotaomicron BFG109* with a 99% match. This was obtained from one of ten PCR-amplified products using universal bacterial primers; (b) Bacterial identification occurred through Sanger sequencing and BLASTn analysis, identifying the sample as *Limosilactobacillus fermentum L4* with a 99% sequence match, based on one of five PCR-amplified products using Lactobacillus-specific primers. Figure S2: Alamar blue cell viability assessment of diet-influenced metabolites. Figure S3: (a) Untargeted integrated total ion chromatograms of *B. thetaiotaomicron* under dietary conditions; (b) Untargeted integrated total ion chromatograms of *L. fermentum* under dietary conditions; (c) Untargeted integrated total ion chromatograms of co-culture under dietary conditions. Figure S4: (a) Western blot images for ET+ metabolites exposed on INS-1 832/3 cells (a) *B. thetaiotaomicrn*, (b) *L. fermentum* and (c) co-culture. (*n* = 3); (b) Western blot images for ET− metabolites (*B. thetaiotaomicron*, *L. fermentum* and co-culture) exposed on INS-1 832/3 cells (*n* = 3).
