# Supplementary material for: Investigating the Role of Diet-Manipulated Gut Bacteria in Pathogenesis of Type 2 Diabetes Mellitus—An In Vitro Approach
Source: Nutrients. 2026 Jan 15;18(2):279. doi: 10.3390/nu18020279 (PMC12844667; doi:10.3390/nu18020279)
Supplement: Supplementary file 1 [file nutrients-18-00279-s001.zip › nutrients-4054692-supplementary.pdf]

Table S1.Integrated peak list of B. thetaiotaomicron common metabolites eluted at similar retention times under control, high-fibre and high carbohydrate/fat conditions.

Potential common metabolites	Control	High-fibre	High carbohydrates/fat	Possible gut bacterial mechanisms associated with the eluted metabolites
Isovaleric acid; butanoic acid, 3-methyl-	4.9	7.8	-	Short-chain fatty acid metabolism (SCFA), derived from amino acid catabolism (Vernocchi et al., 2020; Vidal-Veuthey et al., 2022).
hexanoic acid, 2-methyl-	5.1	9.2	-	Short-chain fatty acid metabolism (SCFA)(Vernocchi et al., 2020)
2 propanioc acid	-	11.2	-	Short-chain fatty acid metabolism (SCFA), derived from amino acid catabolism (Vernocchi et al., 2020; Vidal-Veuthey et al., 2022)
cyclo(L,prolyl-l-valine)	11.9	-	-	Diketopiperazine metabolism and peptide biosynthesis (Bofinger et al., 2017; Kapadia et al., 2022; Ogilvie and Czekster 2023)
Cyclo(leucycloprolyl)	12.1	-	-	Diketopiperazine metabolism and peptide (Bofinger et al., 2017; Kapadia et al., 2022; Ogilvie and Czekster 2023)
hexadecanamide	13.8	13.8	13.8	Fatty acid amide metabolism (Kado 2024; Mueller and Driscoll 2009; Needham, Kaddurah-Daouk and Mazmanian 2020)
9-Octadecenamide, (Z)- or oleamide	14.9	14.9	14.9, 14.98	Fatty acid amide metabolism (Kado 2024; Mueller and Driscoll

				2009; Needham, Kaddurah-Daouk and Mazmanian 2020)
Phenol, 2,2'-methylenebis(6-(1,1-dimethylethyl)-4-methyl-	15.23	15.22	11.38, 15.23	Phenolic compound metabolism and secondary metabolite biosynthesis. (Wang et al., 2022)
Heptacosane	16.45, 16.7,17.4, 18.3, 19.4	15.3, 18.3, 19.4	16.08, 16.72, 16.95, 17.48, 18.14, 18.39, 19.48, 19.55	Alkane metabolism, derived from lipid degradation
Tetratetracontane	-	16.4	14.02	Alkane metabolism, derived from lipid degradation

Table S2.Integrated peak list of L. fermentum common metabolites eluted at similar retention times under control, high-fibre and high carbohydrate/fat conditions.

**Potential common metabolites**	**Control**	**High-fibre**	**High carbohydrates/fat**	**Possible gut bacterial mechanisms associated with the eluted metabolites**
Hexanoic acid, 2-ethyl-butyl ester	4.67	9.41		Short-chain fatty acid metabolism (SCFA), derived from amino acid catabolism (Vernocchi et al., 2020; Vidal-Veuthey et al., 2022)
L-lysine	7.2		10.02	Cadeverine bioamine catabolism (Razquín et al., 2019; Tang et al., 2025)
p-Cymene	8.1			
1-phenathrenecarboxyllic acid	8.39			Carboxylic acid from amino acid catabolism
Cyclohexane carboxylic acid	8.44			Carboxylic acid from amino acid catabolism
D-glucopyranosiduronic acid	9.06			
ethane, 1,2-phenoxy-	11.87	11.873	11.22	
Cyclo L-prolyl-L-Valine	11.94	11.94, 12.07, 12.41		Diketopiperazine metabolism and peptide biosynthesis (Bofinger et al., 2017; Kapadia et al., 2022; Ogilvie and Czekster 2023)
I-Norvaline, npropargyloxycarbonyl-nonyl ester	12.56	8.11		

Hexadecanamide	13.85	13.83		Fatty acid amide metabolism (Kado, 2024; Mueller and Driscoll 2009; Needham, Kaddurah-Daouk and Mazmanian 2020)
Phenol, 2,2'methylenebis(6-(1,1,-dimethylethyl)-4-methyl-	15.07	15.206	15.206	Phenolic compound metabolism and secondary metabolite biosynthesis (Wang et al., 2022)
10'-Apo-B-psi-carotenoic acid, 5,6-dihydro, 5'6-dihydroxy-, methyl ester		10.02		Possible carotenoid degradation pathways
1,4-diazabicyclo(4,3,0)nonan-2,5-dione, 3-methyl		10.644		
Glycyl-l-proline		11.606		Aromatic amino acid degradation
5H-Cyclopentapyrazine, 6-7-dihydro-5-methyl			7.6	
Glycine, N-(4methylbenzoyl)-methyl ester			7.66	
1,3,8,p-Menthatriene			7.94	
lactone			9.58	
2-benzyloxyphenyl acetic acid			12.78	Carboxylic acid from amino acid catabolism

Table S3. Integrated peak list of co-culture common metabolites eluted at similar retention times under control, high-fibre and high carbohydrate/fat conditions.

**Potential common metabolites**	**Control**	**High-fibre**	**High carbohydrates/fat**	**Possible gut bacterial mechanisms associated with the eluted metabolites**
Isovaleric acid; butanoic acid, 3-methyl-	4.779			Short-chain fatty acid metabolism (SCFA), derived from amino acid catabolism (Vernocchi et al., 2020; Vidal-Veuthey et al., 2022)
Hexanoic acid, 2-methyl-	4.961			Short-chain fatty acid metabolism (SCFA), derived from amino acid catabolism (Vernocchi et al., 2020; Vidal-Veuthey et al., 2022)
Glycerin	6.724			Central metabolism (Engels et al., 2016; J. Zhang et al., 2018)
N-methylene-2-phenylethanamine	7.92	7.867		Amino acid metabolism and secondary metabolite biosynthesis
Cyclo(L,prolyl-l-valine)	11.99	11.94		Diketopiperazine metabolism and peptide biosynthesis (Bofinger et al., 2017; Kapadia et al., 2022; Ogilvie and Czekster, 2023)
Hexadecanamide	13.871	13.85		Fatty acid amide metabolism (Kado 2024; Mueller and Driscoll 2009; Needham, Kaddurah-Daouk and Mazmanian 2020)

9-Octadecenamide, (Z)- or oleamide	14.91	14.89		Fatty acid amide metabolism (Kado 2024; Mueller and Driscoll 2009; Needham, Kaddurah-Daouk and Mazmanian 2020)
Phenol, 2,2'-methylenebis(6-(1,1-dimethylethyl)-4-methyl-	15.23	15.2		Phenolic compound metabolism and secondary metabolite biosynthesis (Wang et al., 2022)
1-pentanone,1-(4-methylphenyl)-		7.41, 7.65	7.6, 7.65	Phenolic compound metabolism and secondary metabolite biosynthesis (Wang et al., 2022)
Glycocholic acid		9.32	9.32	Cholate compounds
1,4 Diazabicyclo (4,3.0) nonan-2,5-dione-3-methyl	11.414	11.37		Nitrogen compounds
l-alanine,N-(2-thienylcarbonyl)-, hexyl ester		11.6		Branched chain amino acid catabolism (Ogilvie and Czekster 2023)
ethane-1,2-diphenoxy-		11.87	11.87	
1H-Imidazo(4,5-d) pyridine		7.09		
L-leucine, N-cyclopropylcarbonyl-butyl ester		12.48	12.42	Branched chain amino acid catabolism
1-methyl-2,5-dipropyldecahydroquinoline		12.66	12.67	
Nonanamide		14.96	14.96	Phenolic compound metabolism and secondary metabolite biosynthesis (Wang et al., 2022)

heptanoic acid ethyl ester	9.459		8.39	Carboxylic acid from amino acid catabolism
cyclohexane carboxylic acid, 1-tert-butyl-			8.44	Carboxylic acid from amino acid catabolism
2,3-pyridinedicarbonitrile			8.8	
1,3-propanediol			10.1	Central metabolism (Engels et al., 2016; J. Zhang et al., 2018
cyclo(leucycloprolyl)	-	12.51,<br>12.59	-	Branched chain amino acid catabolism

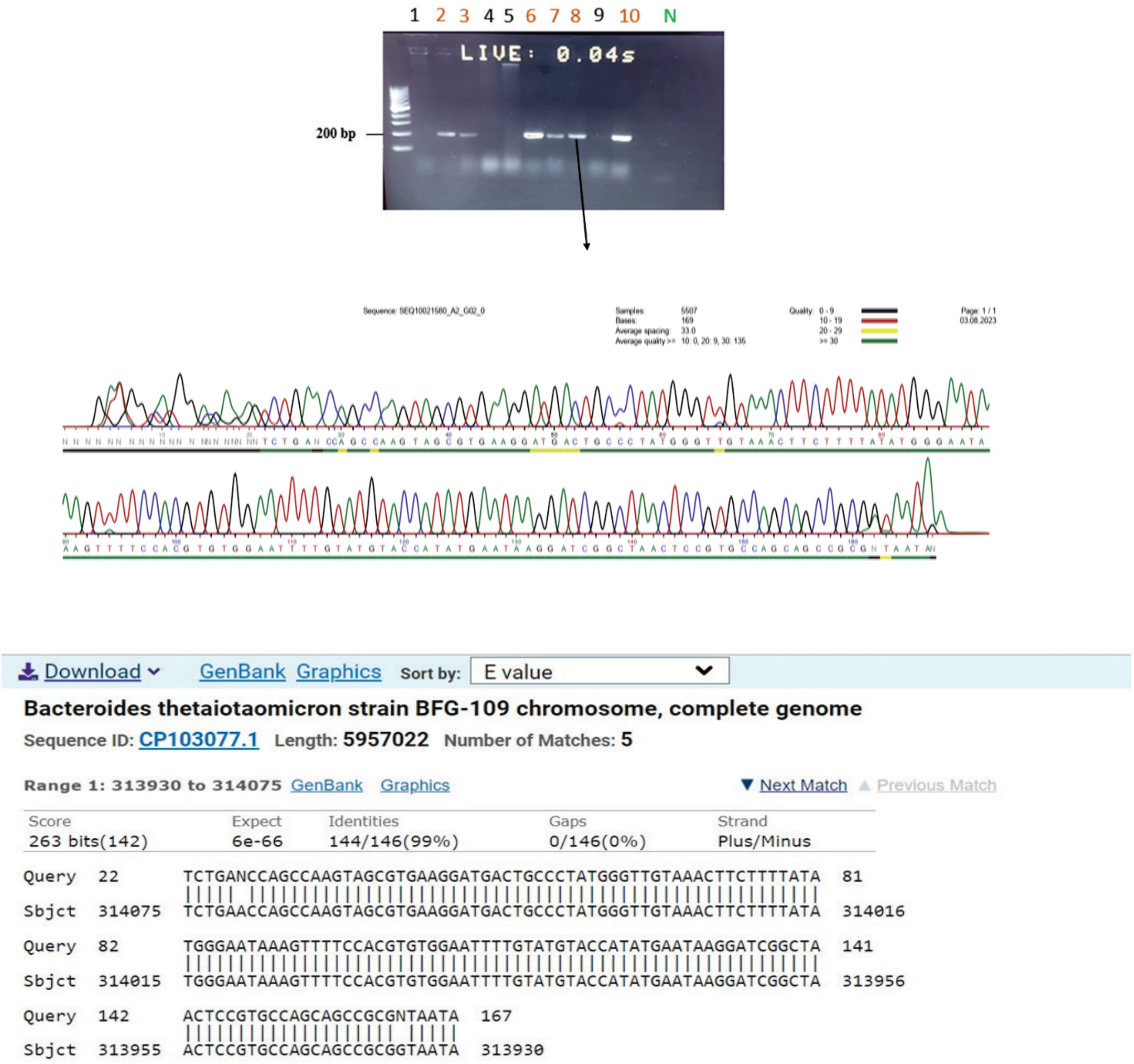

Figure S1a. Bacterial identification via Sanger sequencing and BLASTn analysis revealed *Bacteroides thetaiotaomicron BFG109* with a 99% match. This was obtained from one of ten PCR-amplified products using universal bacterial primers.

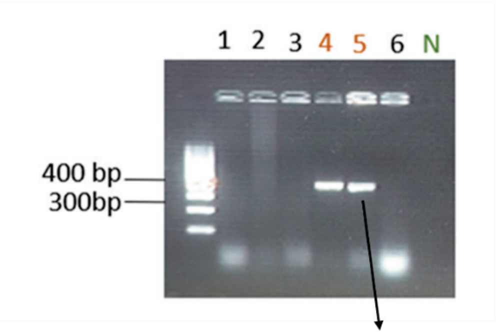

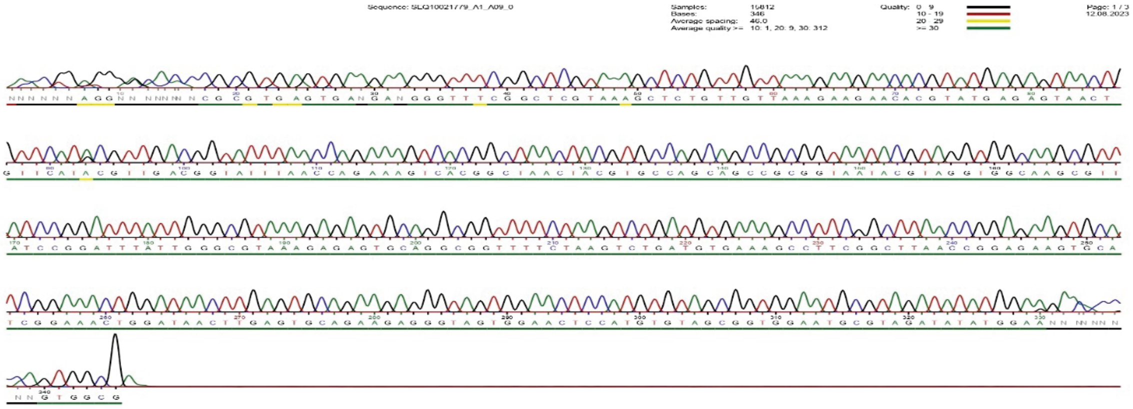

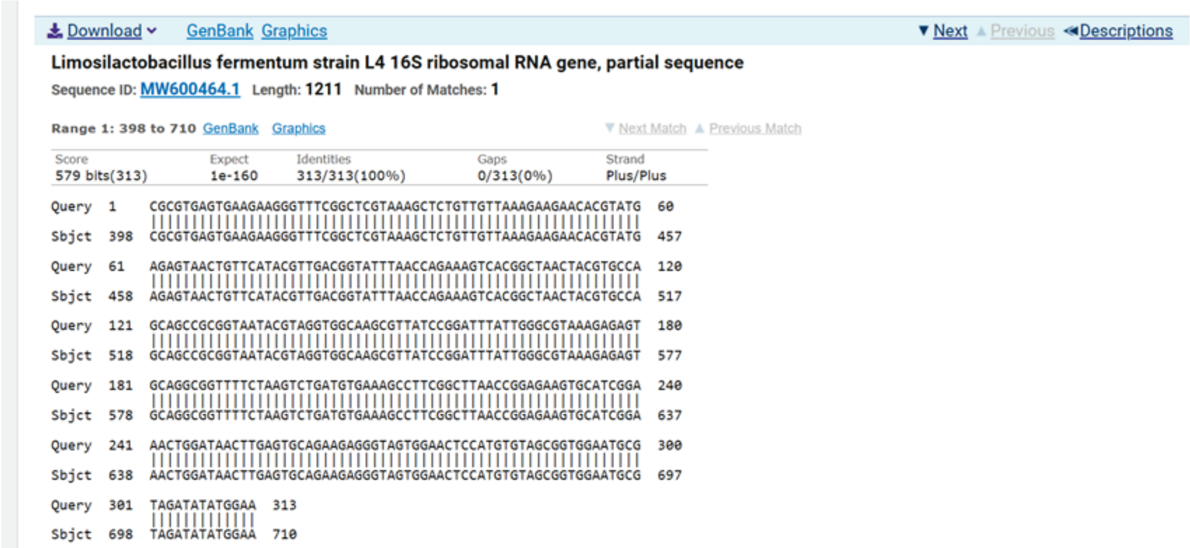

Figure S1b.Bacterial identification occurred through Sanger sequencing and BLASTn analysis, identifying the sample as *Limosilactobacillus fermentum L4* with a 99% sequence match, based on one of five PCR-amplified products using Lactobacillus-specific primers

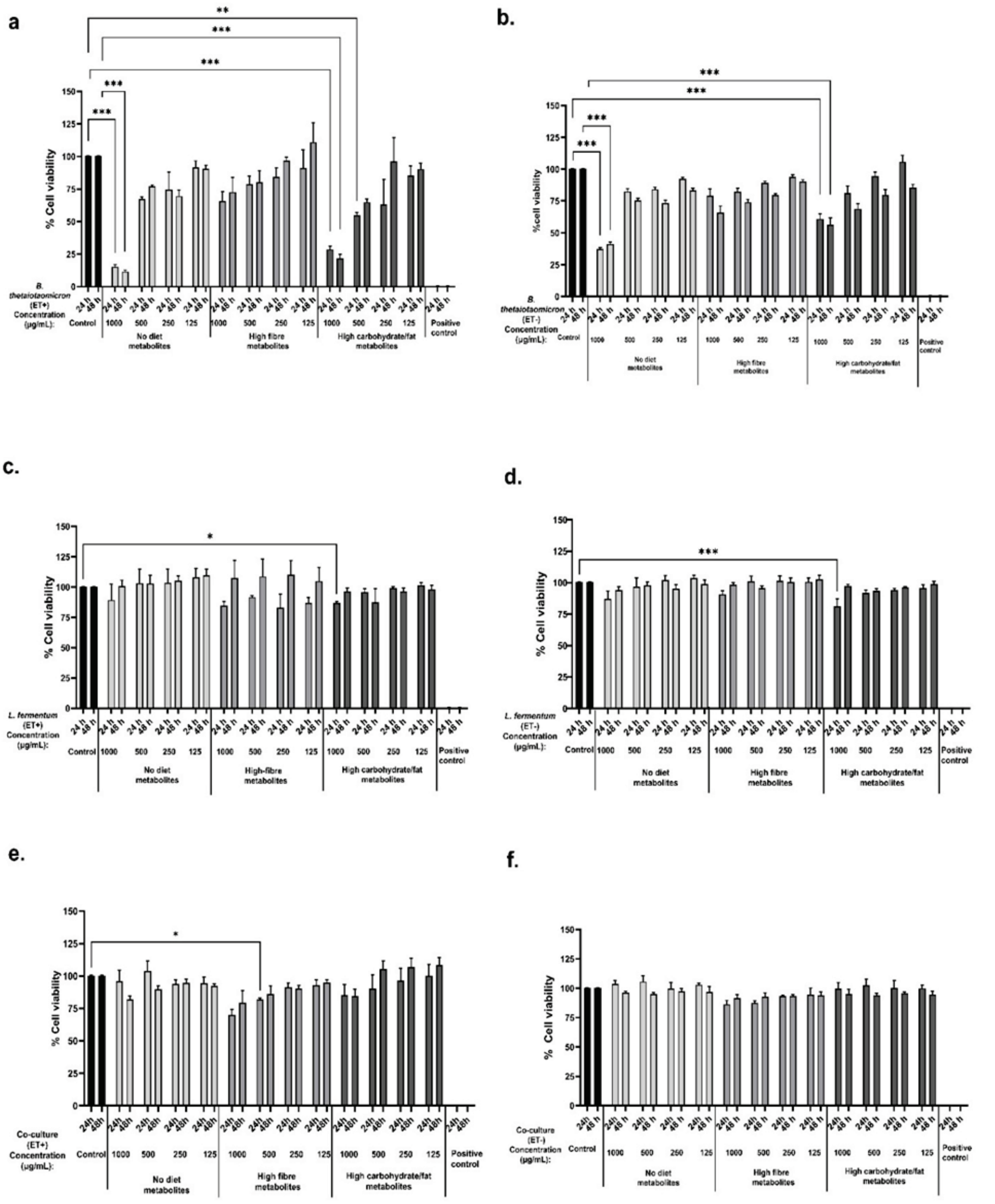

Figure S2. Alamar blue cell viability assessment of diet-influenced metabolites.

The figure illustrates cell viability in response to various diet-influenced metabolites (ET+/-, control or no diet, high-fibre, and high-carbohydrate/fat) at concentrations from 0 to 1000 µg/mL in INS-1 832/3 cells, observed at 24 and 48 h. It includes monocultures of B. thetaiotaomicron (a, b) and L. fermentum (c, d), as well as their co-culture (e, f). The values are depicted as the mean ± SEM (n=3) with significance indicated by *$p < 0.05$, **$p < 0.005$, and ***$p < 0.0005$.

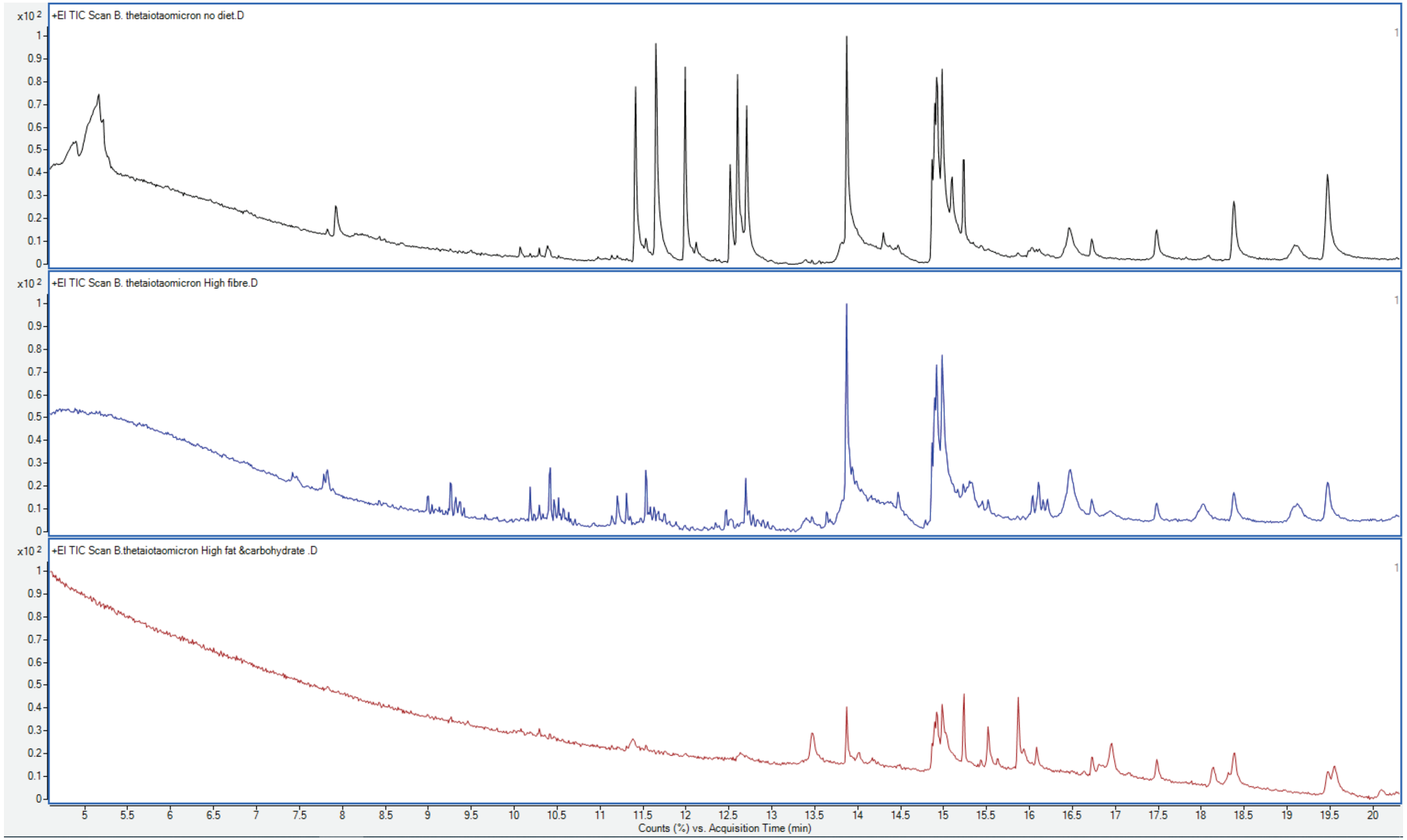

Figure S3a. Untargeted integrated total ion chromatograms of B. thetaiotaomicron under dietary conditions. (Black: no diet; control conditions; Blue: high-fibre; Red: high carbohydrate/fat conditions).

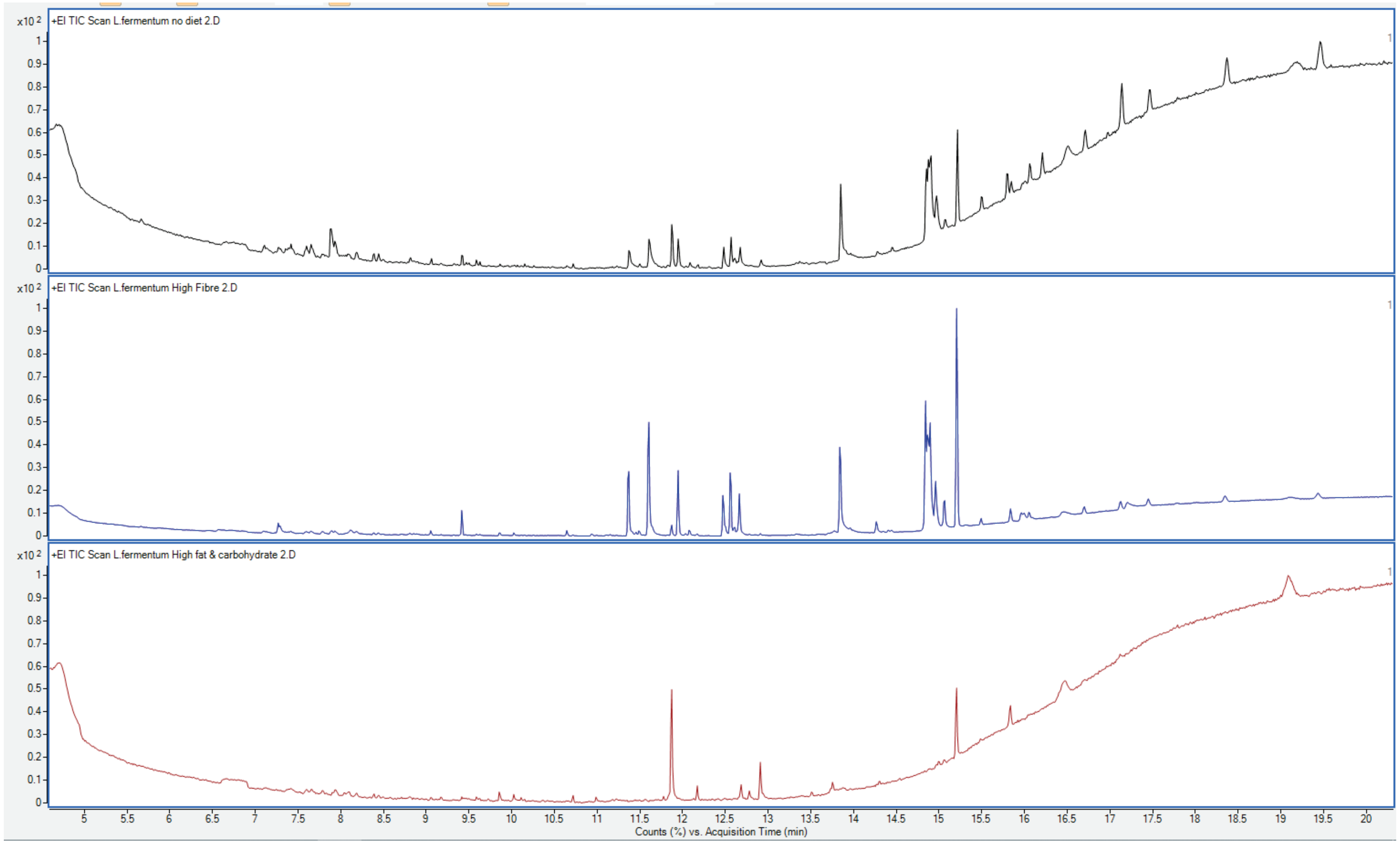

Figure S3b. Untargeted integrated total ion chromatograms of L. fermentum under dietary conditions (Black: no diet; control conditions, Blue: high-fibre, and Red: high carbohydrate/fat conditions).

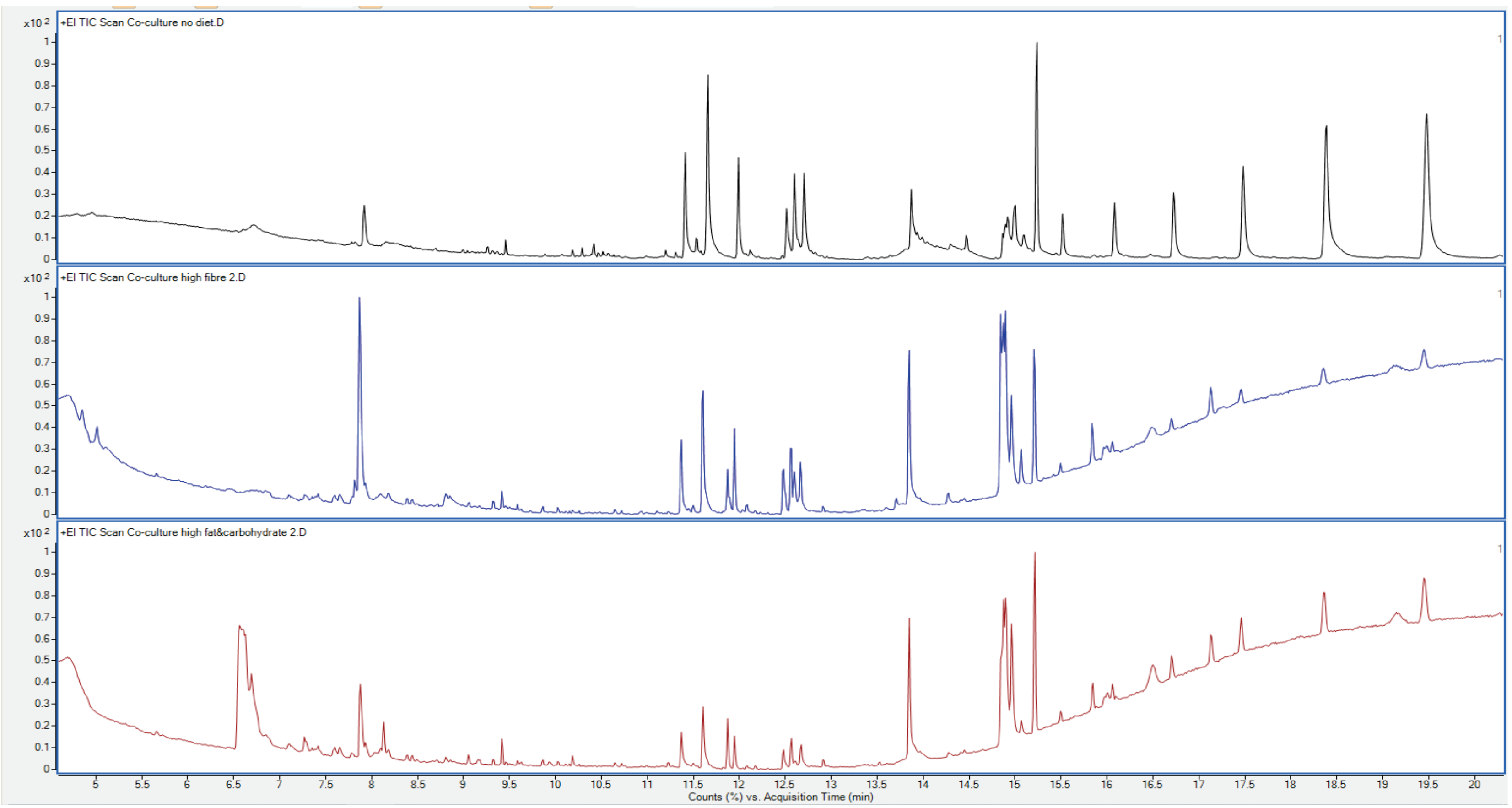

Figure S3c. Untargeted integrated total ion chromatograms of co-culture under dietary conditions (Black: no diet; control conditions, Blue: high-fibre, and Red: high carbohydrate/fat conditions.

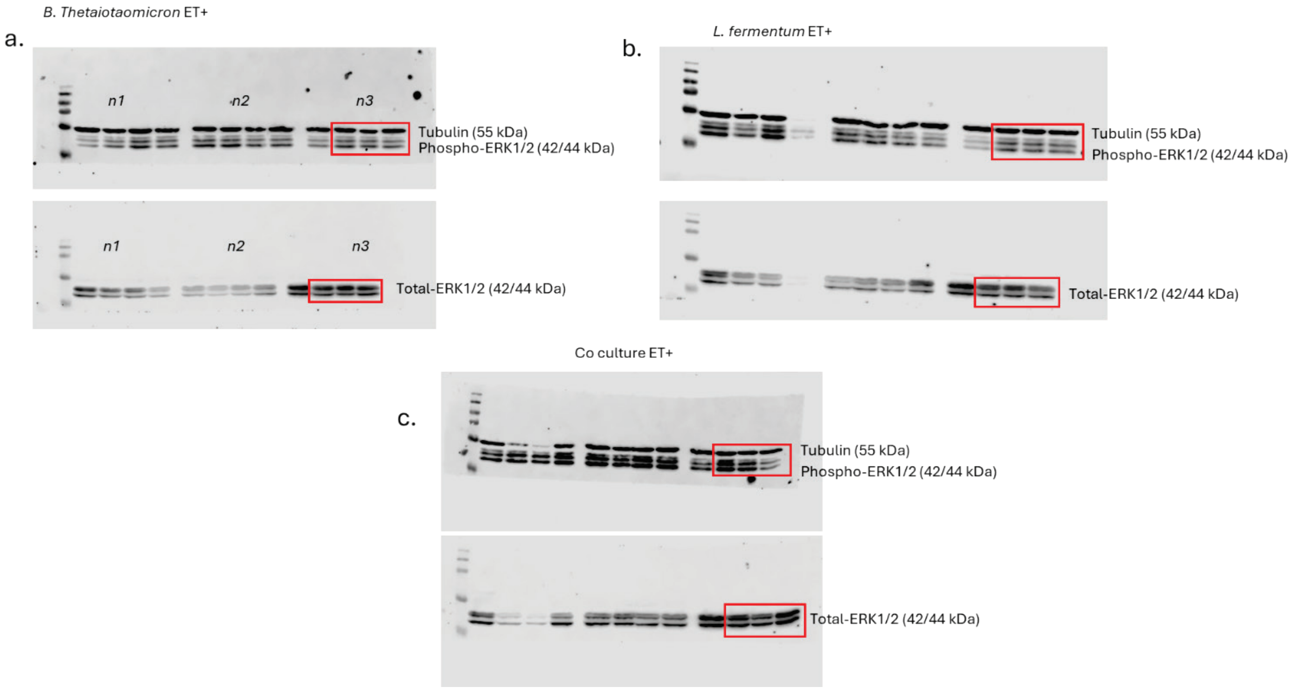

Figure S4a. Western blot images for ET+ metabolites exposed on INS-1 832/3 cells *a. B.thetaiotaomicrn, b. L.fermentum* and c. co-culture. (n=3).

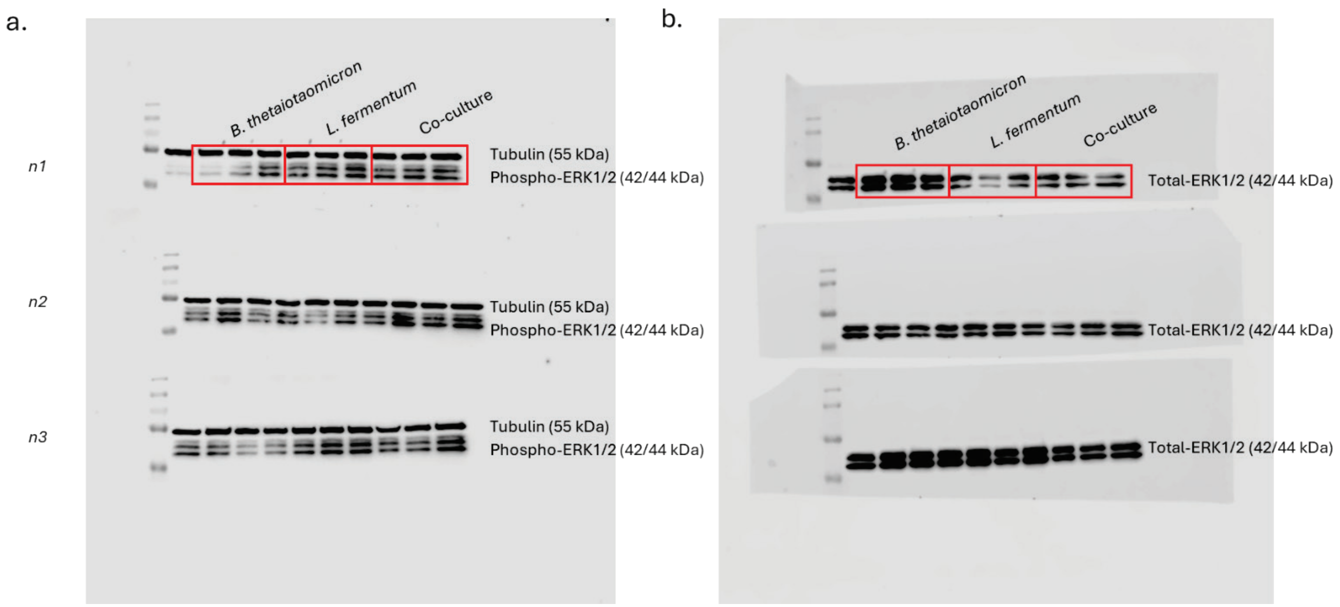

Figure S4b. Western blot images for ET- metabolites (*B.thetaiotaomicron, L.fermentum* and co-culture) exposed on INS-1 832/3 cells (n=3).
